# A Genomic Pathway Approach to a Complex Disease: Axon Guidance and Parkinson Disease

**DOI:** 10.1371/journal.pgen.0030098

**Published:** 2007-06-15

**Authors:** Timothy G Lesnick, Spiridon Papapetropoulos, Deborah C Mash, Jarlath Ffrench-Mullen, Lina Shehadeh, Mariza de Andrade, John R Henley, Walter A Rocca, J. Eric Ahlskog, Demetrius M Maraganore

**Affiliations:** 1 Division of Biostatistics, Department of Health Sciences Research, Mayo Clinic College of Medicine, Rochester, Minnesota, United States of America; 2 Department of Neurology, Miller School of Medicine, University of Miami, Miami, Florida, United States of America; 3 Gene Logic, Gaithersburg, Maryland, United States of America; 4 Molecular and Cellular Pharmacology, Miller School of Medicine, University of Miami, Miami, Florida, United States of America; 5 Department of Physiology and Biomedical Engineering, Mayo Clinic College of Medicine, Rochester, Minnesota, United States of America; 6 Division of Epidemiology, Department of Health Sciences Research, Mayo Clinic College of Medicine, Rochester, Minnesota, United States of America; 7 Department of Neurology, Mayo Clinic College of Medicine, Rochester, Minnesota, United States of America; Baylor College of Medicine, United States of America

## Abstract

While major inroads have been made in identifying the genetic causes of rare Mendelian disorders, little progress has been made in the discovery of common gene variations that predispose to complex diseases. The single gene variants that have been shown to associate reproducibly with complex diseases typically have small effect sizes or attributable risks. However, the joint actions of common gene variants within pathways may play a major role in predisposing to complex diseases (the paradigm of complex genetics). The goal of this study was to determine whether polymorphism in a candidate pathway (axon guidance) predisposed to a complex disease (Parkinson disease [PD]). We mined a whole-genome association dataset and identified single nucleotide polymorphisms (SNPs) that were within axon-guidance pathway genes. We then constructed models of axon-guidance pathway SNPs that predicted three outcomes: PD susceptibility (odds ratio = 90.8, *p* = 4.64 × 10^−38^), survival free of PD (hazards ratio = 19.0, *p* = 5.43 × 10^−48^), and PD age at onset (*R* ^2^ = 0.68, *p* = 1.68 × 10^−51^). By contrast, models constructed from thousands of random selections of genomic SNPs predicted the three PD outcomes poorly. Mining of a second whole-genome association dataset and mining of an expression profiling dataset also supported a role for many axon-guidance pathway genes in PD. These findings could have important implications regarding the pathogenesis of PD. This genomic pathway approach may also offer insights into other complex diseases such as Alzheimer disease, diabetes mellitus, nicotine and alcohol dependence, and several cancers.

## Author Summary

Complex diseases are common disorders that are believed to have many causes. Examples include Alzheimer disease, diabetes mellitus, nicotine and alcohol dependence, and several cancers. This study represents a paradigm shift from single gene to pathway studies of complex diseases. We present the example of Parkinson disease (PD) and a complex array of chemical signals that wires the brain during fetal development (the axon guidance pathway). We mined a dataset that studied hundreds of thousands of DNA variations (single nucleotide polymorphisms [SNPs]) in persons with and without PD and identified SNPs that were assigned to axon-guidance pathway genes. We then identified sets of SNPs that were highly predictive of PD susceptibility, survival free of PD, and age at onset of PD. The effect sizes and the statistical significance observed for the pathway were far greater than for any single gene. We validated our findings for the pathway using a second SNP dataset for PD and also a dataset for PD that studied RNA variations. There is prior evidence that the axon guidance pathway might play a role in other brain disorders (e.g., Alzheimer disease, Tourette syndrome, dyslexia, epilepsy, and schizophrenia). A genomic pathway approach may lead to important breakthroughs for many complex diseases.

## Introduction

Complex diseases occur commonly in the population and are a major source of disability and death worldwide. They are thought to arise from multiple predisposing factors, both genetic and nongenetic, and joint effects of those factors are thought to be of key importance [1,2]. Parkinson disease (PD) serves as an example of a complex disease [3,4]. Other examples include Alzheimer disease, diabetes mellitus, nicotine and alcohol dependence, and several types of cancer [5]. While major inroads have been made in identifying the genetic causes of rare Mendelian disorders, little progress has been made in the discovery of common gene variations that predispose to complex diseases [6,7]. The single gene variants that have been shown to associate reproducibly with complex diseases typically have small effect sizes or attributable risks. However, the joint actions of common gene variants within pathways may play a major role in predisposing to complex diseases (the paradigm of complex genetics), and the discovery of susceptibility genes and pathways may have sizeable public health benefits [8,9].

As early as 1997, experimental studies inferred that genetic variability in the axon guidance pathway was a possible factor contributing to the cause of PD [10]. More recently, a high-resolution, whole-genome association study of PD highlighted the semaphorin 5A gene *(SEMA5A)* as containing the single nucleotide polymorphism (SNP) most significantly associated with PD susceptibility in that study [11]. *SEMA5A* maps to the deletion candidate interval for cri du chat syndrome, which is associated with severe abnormalities in brain development [12]. Semaphorin proteins play an important role in axon guidance and in the development of the mesencephalic dopamine neuron system during embryogenesis [13]. They interact with several other proteins from the axon guidance pathway to provide a complex and dynamic set of cues that either repel (as for the semaphorins) or attract axons toward their synaptic targets [14,15]. Indeed, several axon-guidance pathway proteins (including ephrin and netrin and slit proteins and their ephrin and deleted in colorectal carcinoma and roundabout family receptors) have been shown to also be important for dopamine axonal maintenance, regeneration, and target recognition [16,17]. Several experimental findings from the neurodevelopment literature and our preliminary genetic findings led us to hypothesize that while the main effects of a single gene such as *SEMA5A* may be small and of limited significance, the joint effects of multiple axon-guidance pathway genes may predispose to PD.

## Results

This study employed a genomic pathway approach to determine whether polymorphism in the axon guidance pathway predisposed to PD. Specifically, we employed bioinformatic methods to mine an available whole-genome association dataset for SNPs that were within brain-expressed, axon-guidance pathway genes. We then employed statistical methods to construct models of axon-guidance pathway SNPs that predicted three outcomes: PD susceptibility, survival free of PD, and age at onset of PD. The primary whole-genome association study dataset employed by this study included 443 PD cases and 443 unaffected sibling controls (Tier 1 sample). Details regarding the demographic and clinical characteristics of these participants have been reported previously [11]. The median age at onset of PD among the cases was 61 years (range 31–94). Details regarding the SNP markers genotyped, including call rates, Hardy-Weinberg equilibrium estimations, and regenotyping concordance rates were previously reported [11]. Our bioinformatic methods identified 128 brain-expressed axon-guidance pathway genes, and our SNP dataset included 1,460 SNPs within 117 of those genes, as detailed in Table S1. There were no SNP data for 11 genes.

### PD Susceptibility

**Figure 1:**
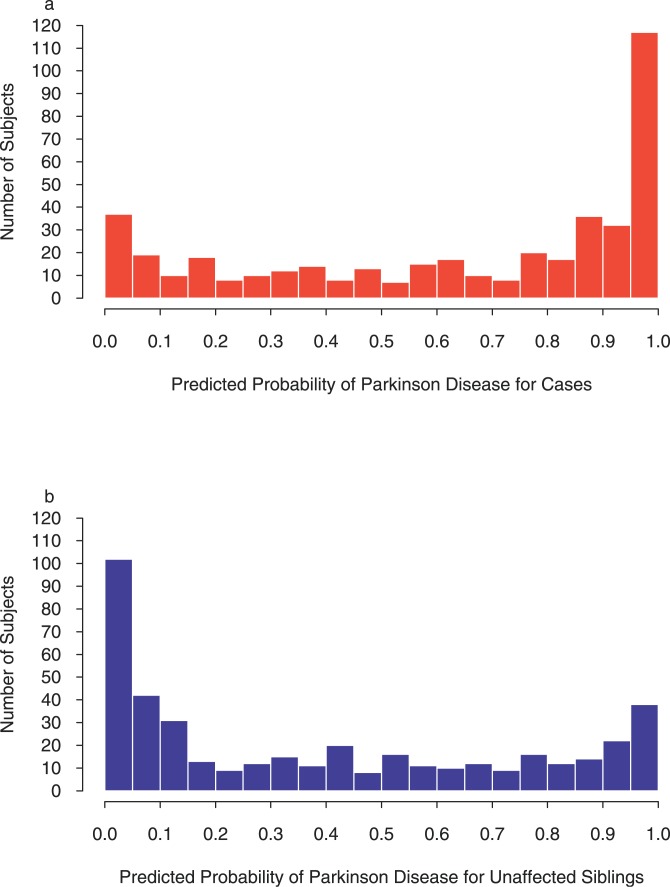
Goodness-of-Fit of Final Model Using Axon Guidance Genes to Predict Susceptibility to PD Data presented are within our primary whole-genome association dataset [11]. Histogram of predicted probabilities of PD in cases (A) and histogram of predicted probabilities of PD in unaffected siblings (B) are presented. A perfect fit would have all predicted probabilities for cases equal to one and all predicted probabilities for unaffected siblings equal to zero. A model with no explanatory value would have histograms that were indistinguishable from each other. Conditional logistic regression analyses were used to generate the probabilities, and hence the data do not fit a single exponential curve (one end close to zero).

Of the 1,460 SNPs within brain-expressed genes of the axon-guidance pathway, 183 SNPs (12.5%) were individually associated with susceptibility to PD. Table 1 contains results for the final model produced by running SNPs through the multistage process to predict PD susceptibility. This model used data from 442 matched PD patients/sibling controls (one pair was missing data on one or more SNPs). The odds ratios (ORs) (95% confidence intervals [CIs]) for the groups defined by predicted PD probability of <0.25, 0.25–0.50, 0.50–0.75, and >0.75 were as follows: 1 (reference), 4.58 (2.27–9.23), 15.42 (6.19–38.45), and 90.76 (32.60–252.67) respectively. Since we were interested in the significance of the pathway, rather than individual SNPs, the *p* value for the overall model was of primary importance. In this case, the model had an overall *p* value of 4.64 × 10^−38^ (95% CI 6.94 × 10^−28^ − 5.39 × 10^−40^). This model significantly predicted whether or not an individual was a case or an unaffected sibling. The predicted probabilities of PD were high (towards 1) for most of the cases, and low (towards 0) for most of the unaffected siblings (Figure 1). Indeed 35% of the cases had predicted probabilities above 0.9, and 34% of unaffected siblings had predicted probabilities below 0.1. However, as shown by Figure 1, the model did not completely distinguish the two groups; some cases had low predicted probabilities, and some controls had high predicted probabilities. The concordance for the model was about 0.70, indicating good but not complete agreement between predicted and observed case/sibling status.

**Table 1:**
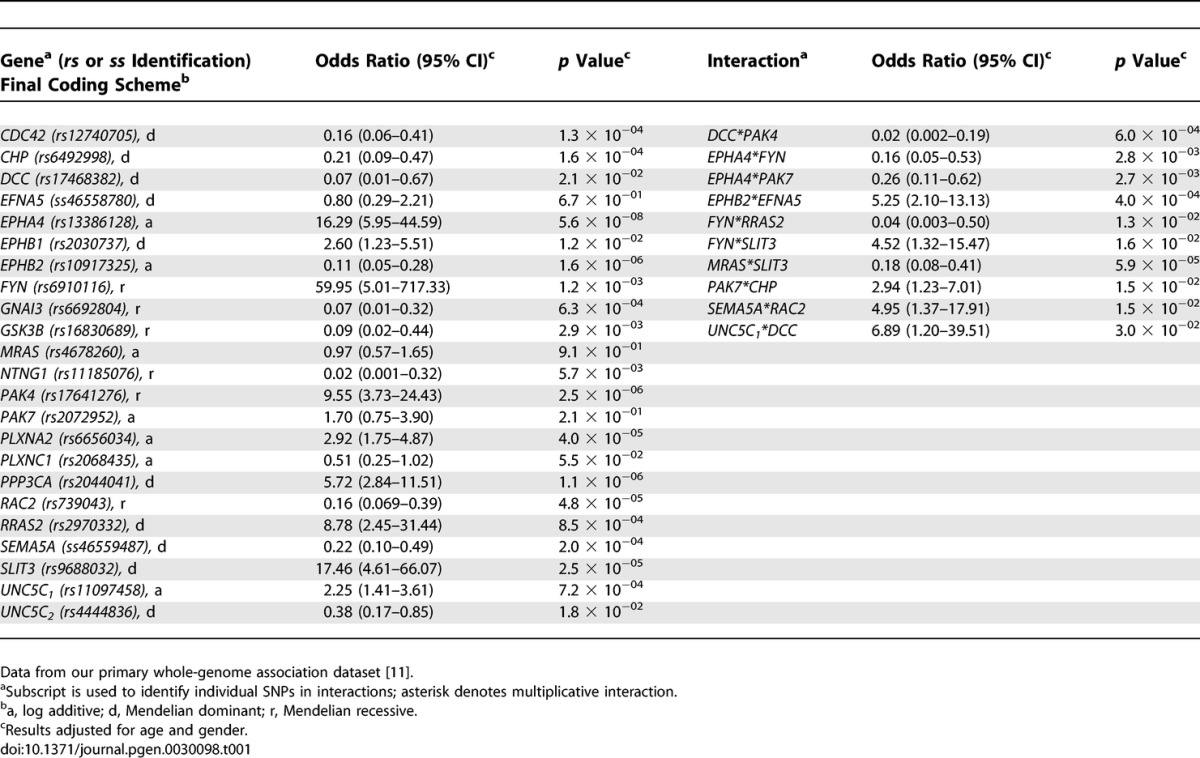
SNPs in Axon-Guidance Pathway Genes Predicting PD Susceptibility

### Survival Free of PD

Of the 1,460 SNPs, 175 (12.0%) were individually associated with survival free of PD (hazard function) using Cox proportional hazards models, as detailed in Table S1. Table 2 contains results for the final proportional hazards model produced by running SNPs through the multistage process to predict survival free of PD. This model used data from 400 PD patients (43 patients were missing data on one or more SNPs). In this case, the model had an overall *p* value of 5.43 × 10^−48^ (95% CI 3.19 × 10^−37^ − 2.36 × 10^−61^). By contrast, the model was not significant at predicting survival (age at study) of the matched sibling controls (*p* = 0.73). This last finding suggests that the model predicts survival free of PD (hazard function), but not survival in general, and that the model is specific for PD cases.

**Table 2:**
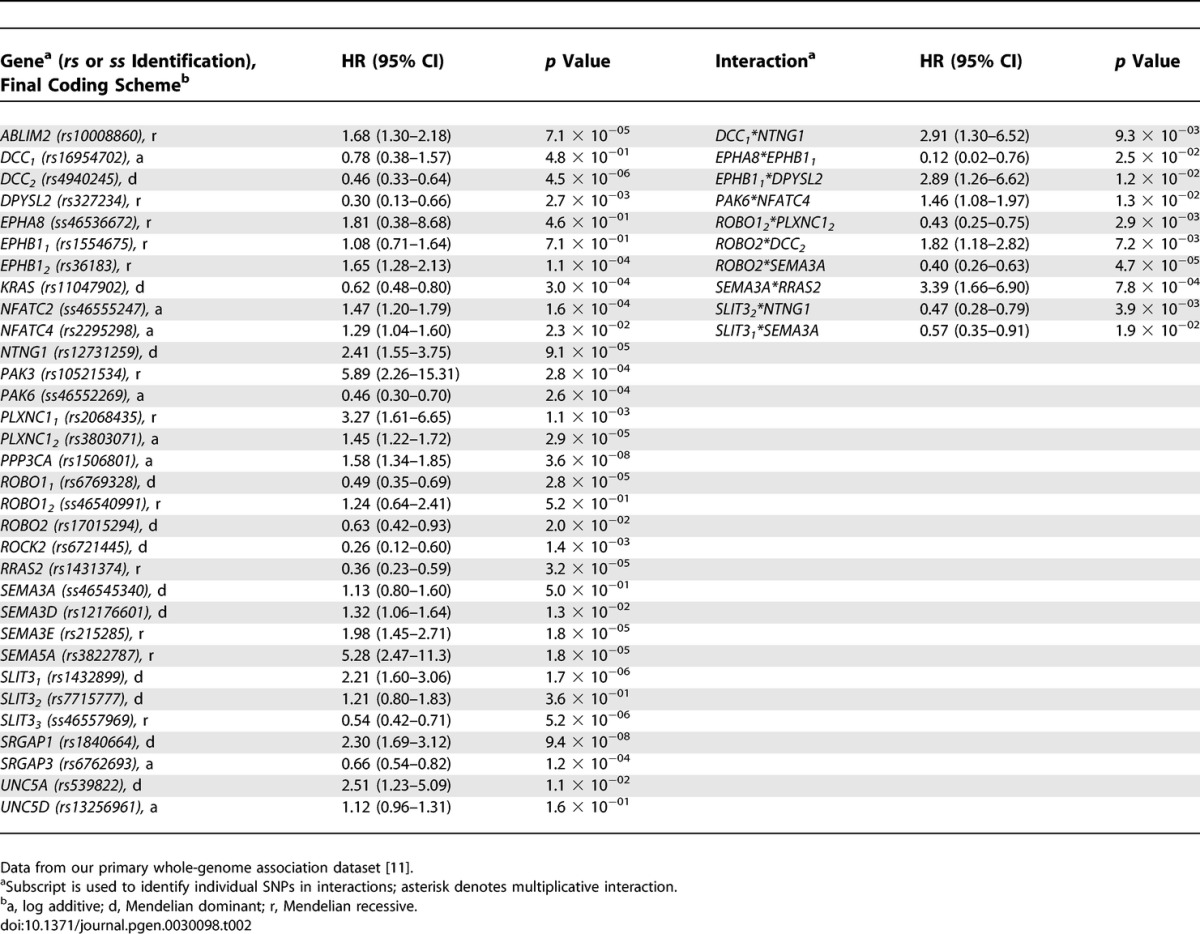
SNPs in Axon-Guidance Pathway Genes Predicting Survival Free of PD

**Figure 2:**
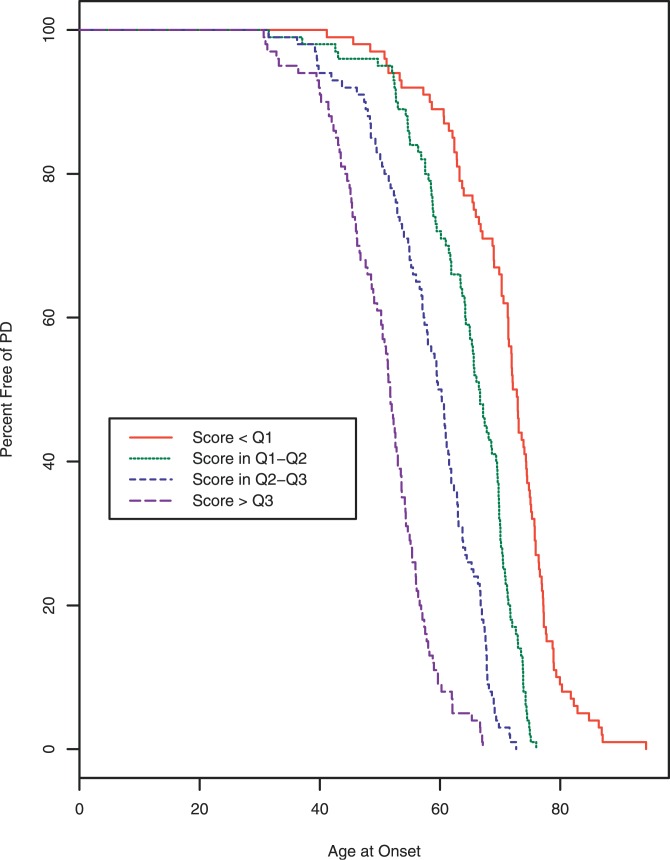
Kaplan-Meier Survival Plot for Age at Onset in PD Patients Data are grouped by categorized risk score from proportional hazards model within our primary whole-genome association dataset [11]. The model clearly differentiates between early-age-at-onset cases and late-age-at-onset cases. Cases generating the long-dashed line were predicted to be at highest risk for early onset of PD, followed by the medium-dashed, short-dashed, and continuous lines, respectively. Since only cases were included, all of the lines end at 0% free of PD.

Figure 2 shows a Kaplan-Meier plot to describe the results of the model. The groups were formed by calculating a risk score for each PD patient using the equation from the proportional hazards model, then categorizing the score at the 25th (Q1), 50th (Q2), and 75th (Q3) percentiles. The survival curves separated nicely right from the earliest ages of onset. By age 60, only 9% of PD patients in the predicted highest risk group were still free of PD, whereas 89% of PD patients in the predicted lowest risk group were free of PD. By age 70, none of the PD patients in the predicted highest risk group were still free of PD, whereas 66% of PD patients in the predicted lowest risk group were free of PD. The median ages at onset for each group, from lowest risk group to highest risk group, were 72.1, 66.5, 59.7, and 51.7, a difference in survival free of PD of more than 20 years from lowest to highest. The concordance for this model was 0.76. The hazards ratios (HRs) (95% CIs) for the four groups, from lowest to highest risk, were 1 (reference), 2.96 (2.14–4.07), 6.91 (4.87–9.81), and 19.04 (13.11–27.65).

### Age at Onset of PD

Of the 1,460 SNPs, 160 (11.0%) were individually associated with age at onset of PD using linear regression models, as detailed in Table S1. Table 3 contains results for the final model produced by running SNPs through the multistage process to predict PD age at onset. This model used data from 395 PD patients (48 patients were missing data on one or more SNPs). In this case, the model had an overall *p* value of 1.68 × 10^−51^ (95% CI 3.56 × 10^−42^ − 1.16 × 10^−51^). By contrast, the set of SNPs was not significant at predicting age at study of the matched sibling controls (*p* = 0.34). This last finding suggests that the model predicts age at onset of PD, not age at the time of the study, and that the model is specific for PD cases.

**Table 3:**
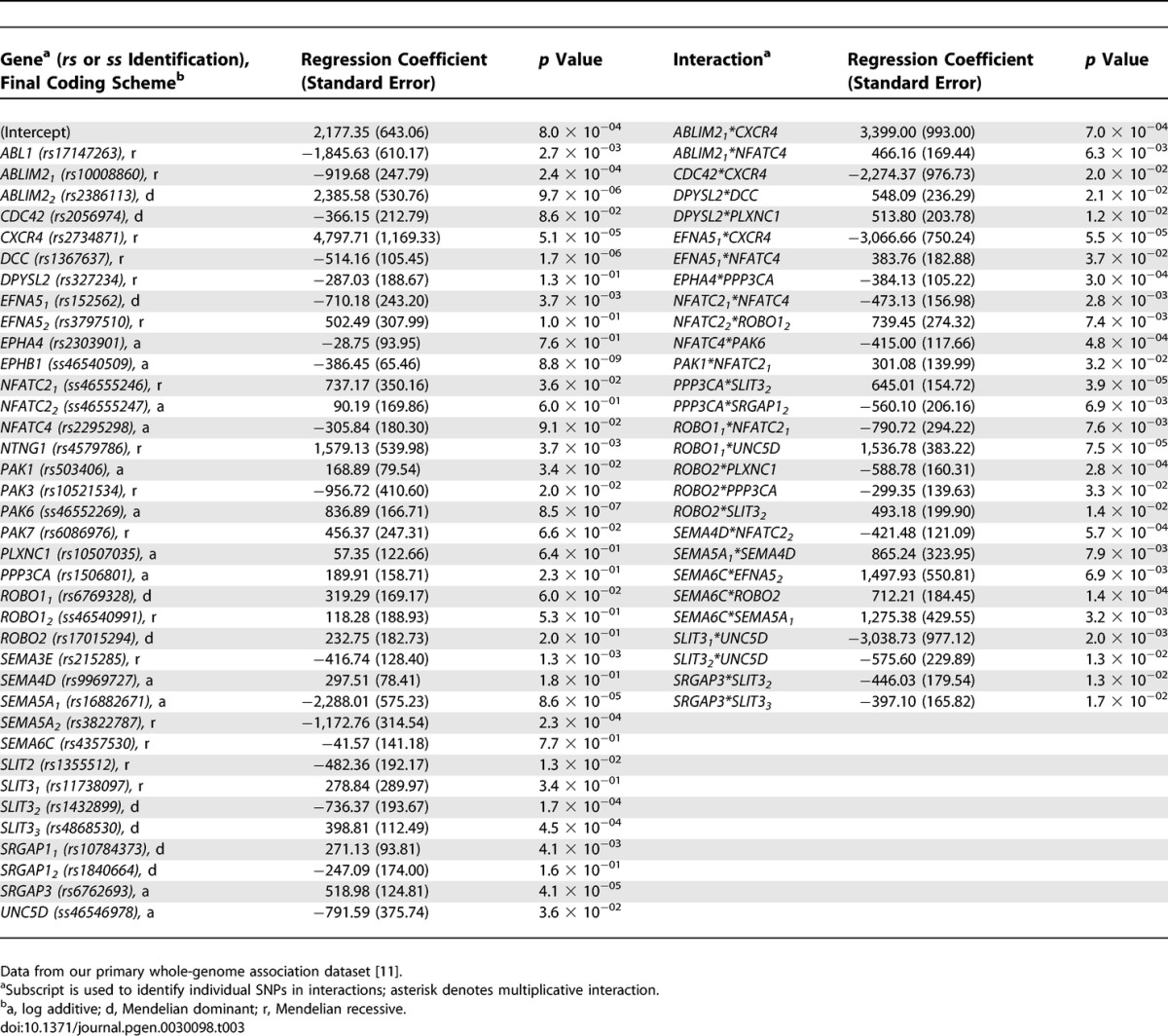
SNPs in Axon-Guidance Pathway Genes Predicting Age at Onset of PD

**Figure 3:**
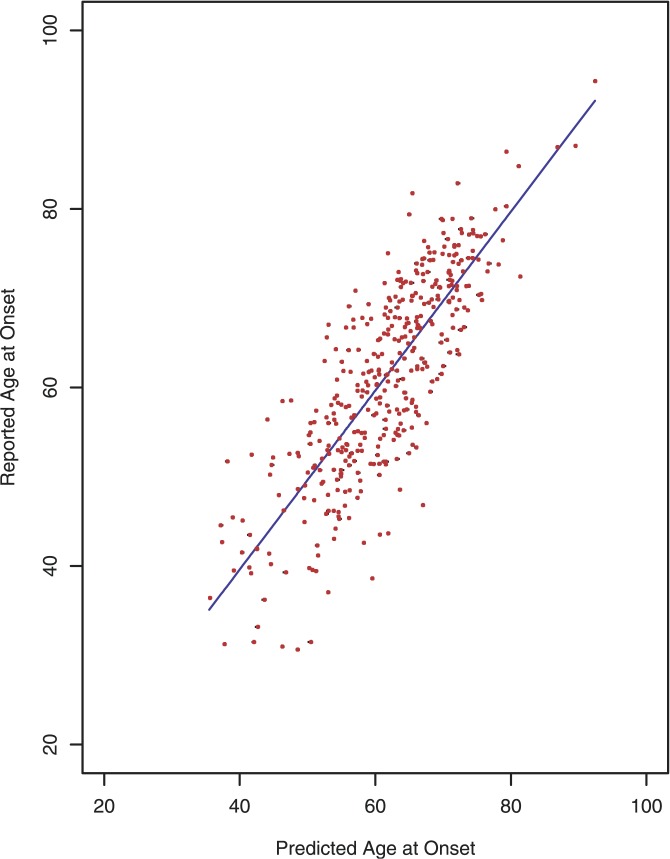
Predicted Versus Reported Age at Onset in PD Patients Data are within our primary whole-genome association dataset [11]. Points indicate reported individual values, and the line represents perfect agreement. In this case, the pattern shows a fairly tight elliptical pattern, reflecting a good fit and high *R* ^2^ of 0.68. The fit appears equally good from the minimum to maximum reported age at onset.

Figure 3 shows a plot of predicted age at onset versus reported age at onset to summarize the results of the model. The plot showed a nice elliptical pattern, reflecting the model *R* ^2^ of 0.683 (95% CI 0.62–0.69). The model explained about 68% of the variability in age at onset of PD.

Figure S1 shows the distributions of the test statistics from the models with randomly selected SNPs and the values of the test statistics from our final models for PD susceptibility (A), survival free of PD (B), and age at onset of PD (C). The test statistics from our final models were in every case much greater than those observed from the other models.

Other combinations of SNPs from the axon guidance pathway also performed quite well in predicting PD susceptibility, survival free of PD, and age at onset of PD. Although the models reported in this manuscript provided good fits to our data, our results do not preclude other combinations of axon-guidance pathway SNPs as significant predictors of PD. The SNPs in the final models that we selected showed no significant linkage disequilibrium in unaffected siblings.

### Validation with a Second Whole-Genome Association Dataset

We also mined a second available whole-genome association dataset for PD, to determine whether the genes in each of the predictive genetic models in our primary whole-genome association dataset were also predictive of the same PD outcomes in the secondary dataset (same genes and outcomes, different SNPs and samples). Details regarding the participants and genotyping procedures for that study have been recently reported [18].

The secondary whole-genome association dataset included 1,195 SNPs in the 22 genes from the model predicting PD susceptibility in our primary dataset. Of those SNPs, 127 (10.6%) were individually associated with susceptibility to PD, as detailed in Table S2. Table S3 contains results for the final model produced by running SNPs through the multistage process to predict PD susceptibility. This model used data from 528 individuals (264 PD patients and 264 unrelated controls; eight individuals were missing data on one or more SNPs). The model had an overall *p* value of 3.93 × 10^−44^. The ORs (95% CIs) for the groups defined by predicted PD probability of <0.25, 0.25–0.50, 0.50–0.75, and >0.75 were as follows: 1 (reference), 7.86 (3.94–15.71), 16.14 (8.13–32.05), and 121.14 (56.63–259.14), respectively. The predicted probabilities of PD were high (towards 1) for most of the cases and low (towards 0) for most of the unaffected siblings (Figure S3). Indeed 38% of the cases had predicted probabilities above 0.9, and 39% of unaffected siblings had predicted probabilities below 0.1. However, as shown by Figure S3, the model did not completely distinguish the two groups; some cases had low predicted probabilities, and some controls had high predicted probabilities. The concordance for the model was 0.90.

The secondary whole-genome association dataset included 1,411 SNPs in the 26 genes from the model predicting age at onset of PD in our primary dataset. Of those SNPs, 142 (10.1%) were individually associated with survival free of PD (hazard function) using Cox proportional hazards models, as detailed in Table S2. Table S4 contains results for the final proportional hazards model produced by running SNPs through the multistage process to predict survival free of PD. This model used data from 263 PD patients (five patients were missing data on one or more SNPs). In this case, the model had an overall *p* value of 6.30 × 10^−35^. However, the model was not significant at predicting survival (age at study) of the matched sibling controls (*p* = 0.14).

Figure S4 uses a Kaplan-Meier plot to describe the results of the model. The groups were formed by calculating a risk score for each PD patient using the equation from the proportional hazards model, then categorizing the score at the 25th (Q1), 50th (Q2), and 75th (Q3) percentiles. The survival curves separated nicely right from the earliest ages of onset. By age 60, only 41% of PD patients in the predicted highest risk group were still free of PD, whereas 94% of PD patients in the predicted lowest risk group were free of PD. By age 70, none of the PD patients in the predicted highest risk group were still free of PD, whereas 68% of PD patients in the predicted lowest risk group were free of PD. The median ages at onset for each group, from lowest risk group to highest risk group, were 74, 68, 63, and 60, a difference in survival free of PD of 14 years from lowest to highest. The concordance for this model was 0.78. The HRs (95% CIs) for the four groups, from lowest to highest risk, were 1 (reference), 4.02 (2.62–6.16), 10.69 (6.72–16.98), and 21.05 (12.85–34.48).

The secondary whole-genome association dataset included 1,605 SNPs in 28 of the 29 genes from the final model predicting age at onset of PD in our primary dataset. Of those SNPs, 157 (9.8%) were individually associated with age at onset of PD using linear regression models, as detailed in Table S2. Table S5 contains results for the final model produced by running SNPs through the multistage process to predict PD age at onset-squared. This model used data from 265 PD patients (three patients were missing data on one or more SNPs). In this case, the model had an overall *p* value of 4.72 × 10^−40^. However, the set of SNPs was not significant at predicting age at study-squared of the matched sibling controls (*p* = 0.527).

Figure S5 shows a plot of predicted age at onset versus reported age at onset to summarize the results of the model. The plot showed a nice elliptical pattern, reflecting the model *R* ^2^ of 0.714. The model explained about 71% of the variability in age at onset of PD.

### Validation with a Gene-Expression Profiling Dataset

**Figure 4:**
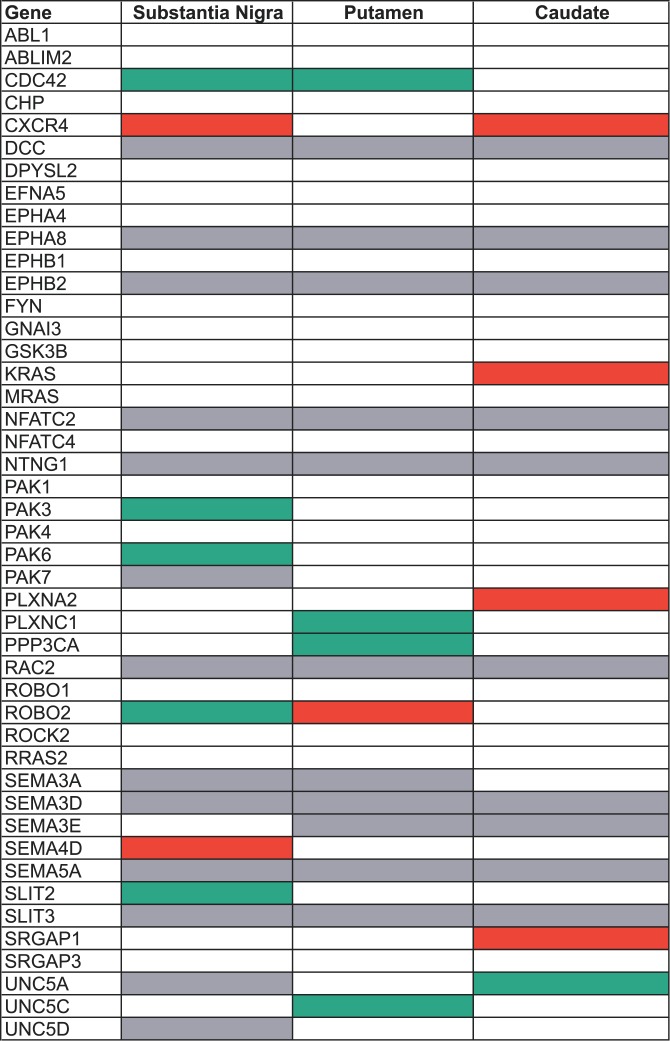
Convergence of Findings for Whole-Genome Association and Gene-Expression Profiling Datasets Listed are genes that had one or more SNPs included in at least one of the three genetic models that predicted PD susceptibility or survival free of PD or age at onset of PD (*n* = 45 genes). Expression data are summarized for each of the three nigrostriatal regions (substantia nigra, putamen, and caudate). Gray, no informative probe sets for the gene and region; white, no difference in expression in cases and controls; red, the gene had increased expression in cases compared to controls; green, the gene had reduced expression in cases compared to controls.

We also mined an available gene-expression profiling dataset for PD that considered 21 different brain regions. Details regarding the participants, biological samples, and microarray experiments have been recently reported [19]. For this study, we limited our data analyses to the substantia nigra and the striatum (putamen and caudate nuclei), since these are the brain regions contributing most significantly to the nigrostriatal dopamine deficiency that is characteristic of PD and since we defined our three PD outcomes according to the corresponding motor phenotype. There was a total of 45 genes represented by the SNPs listed for the three predictive genetic models (Tables 1–3), and the gene expression dataset had informative probe sets for 32 of those genes in the substantia nigra, 34 in the putamen, and 35 in the caudate. Table S6 provides a detailed listing of the multiregional expression data for the 45 genes. In each region, there were more differentially expressed genes observed than expected by chance: substantia nigra, seven observed (22%) versus 1.6 expected (5%); putamen, five observed (15%) versus 1.7 expected (5%); and caudate, five observed (14%) versus 1.8 expected (5%). Overall, 36 genes had data in at least one of the three regions, and 14 (39%) of those were differentially expressed in at least one region. Figure 4 provides a detailed summary of the multiregional expression data for the 45 axon-guidance pathway genes that were predictive of PD outcomes. We observed no significant differences in the number of probe sets for differentially expressed versus normally expressed genes, except for the putamen where two genes with eight fragments each were both differentially expressed (unpublished data). Removing those two genes (outliers) removed the significance (no differences in the median numbers of informative probe sets).

We found very similar results from our sensitivity analyses treating accurate-type and cross-reacting type fragments equally. Again, in each region there were more differentially expressed genes than expected by chance. Restricting to the 45 genes represented in the three predictive genetic models, the results were: substantia nigra, seven observed (22%) versus 1.6 expected (5%); putamen, five observed (15%) versus 1.7 expected (5%); and caudate, seven observed (20%) versus 1.8 expected (5%). Overall, of the 36 genes with data in at least one of the three regions, 14 (39%) were still differentially expressed in at least one region.

Finally, we renormalized and reanalyzed the raw data from the original gene expression profiling study using a different standard software package, to obtain results for all probe sets in all 45 genes and in all three regions. We again observed differential expression of axon-guidance pathway genes in PD, although the findings were more modest. A total of 13 genes were differentially expressed in at least one region (eight in the substantia nigra, one in the putamen, and four in the caudate).

For all analyses of differential gene expression, we were able to code the differential expression unambiguously as increased or reduced. For genes with multiple informative and differentially expressed probe sets, the direction of effect was always the same.

## Discussion

Although the genetic model findings from this study have not been replicated using identical SNPs in an independent sample, they do generate some interesting hypotheses and provide evidence for the possible role of the axon guidance pathway in PD. As such, our study represents a paradigm shift from single-gene to pathway analyses of a complex disease. Replication of genetic association findings can be challenging, particularly when the effect sizes are small [18, 20–27]. Although a second whole-genome association study of PD was recently published and has data that could be similarly mined to test the hypothesis that the axon guidance pathway is associated with PD, that dataset included only 16% of the SNPs included in the dataset that we utilized, precluding an exact replication of our models [11,18]. Nevertheless, for the 32,192 SNPs common to the two studies, we identified three SNPs that were associated with PD in all meta-analysis strategies. One of the SNPs was in the axon-guidance pathway gene *UNC5C,* which encodes a netrin receptor [28].

Furthermore, within the secondary whole-genome association dataset [18], we were able to construct predictive models for PD susceptibility, survival free of PD, and predicted age at onset of PD. All three models in the secondary dataset were also highly significant. This is remarkable, as the two datasets employed independent samples and different study designs. Namely, the study of Fung et al. employed fewer participants, unrelated controls, and more and different SNP markers compared to the study of Maraganore et al. [11,18]. This would further attest to the robustness of our predictive models. For validation purposes, we restricted the analyses of the secondary dataset to the genes that were represented by SNPs in each of the three predictive models for the primary dataset. This biases the findings for the secondary dataset conservatively, as it is possible that additional axon-guidance pathway genes contribute to the PD outcomes in that study sample. A more conservative approach would have been to limit the analysis of the secondary dataset to either the same SNPs or to SNPs from the same linkage disequilibrium bins, as for the first study. However, because there was only limited overlap in SNPs between the two studies (16%), and because many of the SNPs employed by the two studies are not included in a common haplotype map, this would not be possible for all of the genes in the models. Nevertheless, future studies of the two whole-genome association datasets may wish to consider additional meta-analytic strategies [28].

We note that the magnitudes of the genetic effects were sizeable. Within our primary whole-genome association dataset [11], persons with high versus low model scores differed in susceptibility by 90-fold and in age at onset by more than 20 years, and polymorphism in the axon guidance pathway accounted for nearly 70% of the age at onset variance of PD. Similarly large effects were observed for the secondary dataset [18]. The findings of a study are more likely to be true when the effect sizes are large; however, we acknowledge that limited sample size, multiple tested relationships, and flexible analyses may have reduced the positive predictive value of the large effects that we observed [29]. Although the *p* values observed for our predictive models were small, these were the values determined by the actual data in independent datasets, and bootstrapping resulted in CIs smaller than 1 × 10^−27^ for each outcome in the primary dataset.

Within our primary whole-genome association dataset [11], models constructed from thousands of random selections of genomic SNPs predicted all three PD outcomes poorly by comparison to models constructed using axon guidance SNPs. Our models of survival free of PD and age at onset of PD did not predict age at study in sibling controls (nor for unrelated controls in the secondary dataset). This would again argue against a chance association of the models with PD outcomes within the samples. We note that the models constructed using axon guidance SNPs were robust and were not crippled by the elimination of SNPs. For example, in our primary dataset, even after removing the first ten SNPs to enter the models (via the stepwise selection process), the overall model *p* values were smaller than 1 × 10^−15^ for each of the outcomes (unpublished data). This indicates that the results were not driven by a few SNPs and that the joint effects of multiple SNPs were important.

Our analyses of an available gene-expression profiling dataset revealed that axon-guidance pathway genes were differentially expressed in the brains of PD cases as compared to controls, including several genes that were predictive of PD in the models constructed from the primary whole-genome association dataset (convergence of findings) [11,19]. Not all genes included in the models constructed from the primary whole-genome association dataset were abnormally expressed in the gene-expression profiling dataset, and not all axon-guidance pathway genes that were abnormally expressed in the gene-expression profiling dataset were predictive of PD outcomes in that whole-genome association dataset (unpublished data). This may be because: (1) for some genes, the whole-genome association dataset had no data (no informative SNPs); (2) for some genes, the gene-expression profiling dataset had no data (no informative probe sets); (3) the PD brains included in the gene-expression profiling dataset were from end-stage patients, and it is possible that for some axon-guidance pathway genes differential expression occurs only earlier in life; (4) the genes included in the predictive models may have been associated with PD via a mechanism other than differential expression (i.e., qualitative rather than quantitative effects); (5) there are multiple other possible limitations intrinsic to the whole-genome association and gene expression profiling studies, which may have led to false positive and false negative results (divergence of findings) [11,19].

Although we observed differential expression of axon-guidance pathway genes in an available gene-expression profiling dataset, we have not as yet demonstrated the biological effects of specific SNPs included in the predictive models in either human gene transcription or translation datasets or in experimental cell or animal models (construct validity). We emphasize that the SNPs that were independent predictors of susceptibility, survival free of PD, or age at onset of PD in either whole-genome association dataset may be only indirect markers of the functional variants within the genes. Fine mapping of these gene loci in diverse populations and experimental studies in transfected cell lines or in transgenic animals may help to identify functional variants within the genes and to elucidate the pathogenic mechanism of the axon-guidance pathway variability that we observed [7]. It would be of interest to genotype a large postmortem cohort for the SNPs that were predictive of PD outcomes and to directly correlate the ORs of those SNPs to the fold changes for matching RNA fragments (probe sets).

We find it plausible that polymorphism in the axon-guidance pathway genes could lead to aberrant trajectory of the ascending dopaminergic pathway during embryonic brain development and in turn cause congenital deficiency in nigrostriatal dopamine. In other words, some persons could be “wired differently” from birth and could thus be at greater risk to develop PD and at an earlier age. Animal experiments provide support for this pathogenic mechanism. The study of embryonic brain development in knockout mice has suggested a critical role for axon guidance proteins, including semaphorin and slit proteins, in the guidance of the ascending trajectory of dopamine pathways from cell bodies in the substantia nigra to the ipsilateral striatum [13,30,31]. By the 16th day of embryogenesis, mutant mice deficient in semaphorin and slit proteins exhibit a loss of midline repulsion of ascending dopamine pathways, so that the fibers ascend aberrantly to the contralateral striatum. This results in a visible reduction in the dopamine innervation of the striatum in semaphorin and slit deficient mice. Striatal dopamine deficiency is the neurochemical hallmark of PD [32].

Similarly, transgenic mice with disrupted ephrin signaling exhibit a 40%–50% quantitative reduction in nigrostriatal innervation and exhibit classical behavioral model characteristics of PD [33,34]. There is also some indirect support of this pathogenic “miswiring” mechanism in humans. Recently, a rare sequence variation in a single axonal-dendritic development gene was associated with Tourette syndrome, another disorder involving dopamine neurons [35]. Furthermore, developmental anatomic asymmetries in nigrostriatal innervation resulting from polymorphism in axon-guidance pathway genes could account for motor asymmetries later seen in patients with PD [36].

Another possibility is that axon-guidance pathway molecules contribute to the pathogenesis of PD via other mechanisms. For example, these proteins also play an important role in axonal repair and in apoptotic signaling. Polymorphism within the pathway may make neurons more or less vulnerable to endogenous or exogenous toxins that trigger the cell death response [37,38]. The continued expression of some axon-guidance pathway genes, such as *netrin-1* and *dcc,* in nigral and striatal neurons throughout the mammalian life cycle (including embryogenesis and adulthood) would support multiple possible functions, and it has been suggested that netrin or netrin-like molecules may be of use in understanding and treating PD [10]. Prior knowledge of many biological mechanisms by which the axon guidance pathway may contribute to the pathogenesis of PD increases the probability that our genetic findings may be true (warranting validation studies) [29,39].

### Significance and Innovation

In summary, we employed a genomic pathway approach to determine whether polymorphism in a candidate pathway (axon guidance) predisposed to a complex disease (PD). We found that multiple SNPs in axon-guidance pathway genes were strong predictors of PD susceptibility, survival free of PD, and age at onset of PD in two independent whole-genome association datasets; and many axon-guidance pathway genes were differentially expressed in PD. Although an exact replication of the predictive genetic models remains to be done (same SNPs, independent samples), we consider these findings timely and potentially important. To date, predictive biomarkers for PD are lacking. Our genetic findings for the axon guidance pathway and PD might suggest a new environmental focus on exposures that occur during intrauterine life (miswiring hypothesis).

Our findings would also provide evidence that complex diseases such as PD can be due to the joint effects of many genes that, taken singly, would show only small effects (additive effects model and epistasis) [40–43]. While the available datasets were exploratory and may have been underpowered to detect the main effects of single SNPs or probe sets, our findings are consistent with recent studies that report greater statistical power to detect the joint effects of multiple loci than the main effects of single loci [44,45]. By contrast to familial aggregation and twin studies, our findings demonstrate that a largely sporadic disease such as PD can in fact have a strong genetic component [4,46,47] and suggest that a similar genomic pathway approach might provide insights into several other complex diseases. Finally, these findings provide important insights regarding the molecular mechanisms that may control dopamine circuit formation and programmed cell death in healthy versus diseased individuals. This in turn may facilitate the development of treatments for axonal synaptic regeneration and repair and for neuroprotection in PD [17,37]. We envision that a systematic study of all defined genomic pathways will yield additional important findings for PD, even in the absence of prior evidence of strong single-gene association.

## Materials and Methods

### Bioinformatic methods.

To formally test our hypothesis, we consulted the Kyoto Encyclopedia of Genes and Genomes (KEGG) [48–50]. The KEGG PATHWAY database is a bioinformatics resource that provides wiring diagrams of molecular interactions, reactions, and relations. There are at least 270 pathways in KEGG related to Homo sapiens and diseases. This includes a detailed summary of the axon guidance pathway, updated as recently as October 3, 2005 (http://www.genome.jp/dbget-bin/www_bget?path:hsa04360). We identified all of the genes that encoded proteins within the KEGG axon guidance pathway via Entrez Gene (http://www.ncbi.nlm.nih.gov/entrez/query.fcgi?db=Gene) and consulted the UniGene database (http://www.ncbi.nlm.nih.gov/entrez/query.fcgi?db=unigene) to determine which of the genes were expressed in the human brain (*n* = 128). We then mined an available whole-genome association dataset for PD to identify those SNPs that were genotyped in brain-expressed, axon-guidance pathway genes as part of that study [11]. Specifically, in that study, (which we refer to as the primary whole-genome association dataset), we had individually genotyped 198,345 genomic SNPs that were uniformly spaced (one per 12 kb average gap distance) and informative in 443 sibling pairs discordant for PD. This included 1,460 SNPs within 117 axon-guidance pathway genes expressed in the brain.

### Statistical methods.

All statistical tests were two tailed and considered significant at the conventional alpha level of 0.05. All statistical analyses were performed in SAS version 9.1 (SAS Institute, http://www.sas.com) or S-Plus version 7 (Insightful Corporation, http://www.insightful.com). We considered three outcomes of interest: (1) PD susceptibility, (2) survival free of PD, and (3) age at onset of PD. We sought to identify joint action models of SNPs from the axon guidance pathway that predicted each of the three outcomes.

For the first outcome, we used conditional logistic regressions stratified on sibship to examine associations of the SNPs with PD susceptibility while adjusting for age and gender [51]. For each SNP, we calculated ORs, 95% CIs, and *p* values. Goodness-of-fit was assessed through measuring concordance and visually through histograms of predicted probabilities [52]. We estimated overall ORs by categorizing the predicted probability of PD from the model into four groups (<0.25, 0.25–0.50, 0.50–0.75, and >0.75) and then calculating the ORs for each group relative to the <0.25 group. We used a likelihood ratio test to assess the significance of the overall model and calculated a 95% bias-corrected bootstrap CI for the associated *p* value using 10,000 resamples.

For the second outcome, we used Cox proportional hazards models to test for associations of the SNPs with survival free of PD [53]. For each SNP, we calculated HRs, 95% CIs, and *p* values. Concordance was again calculated for the proportional hazards models, and Kaplan-Meier plots of categorized scores predicting risk of PD were generated to provide visual gauges for goodness-of-fit [54]. We also calculated HRs for risk groups categorized at the quartiles, using the lowest risk group as reference. We used a likelihood ratio test to assess the significance of the overall model and calculated a 95% bias-corrected bootstrap CI for the associated *p* value using 10,000 resamples.

For the third outcome, we predicted the reported age at onset of PD using multiple regression models [55]. Goodness-of-fit was described through the model *R* ^2^ values and plots of the predicted versus observed ages at onset. We used an *F* test to assess the significance of the overall model and calculated a 95% bias-corrected bootstrap CI for the associated *p* value and the *R* ^2^ using 10,000 resamples. Assumptions were tested throughout. Only the linear regression models required a data transformation; age at onset-squared was used as the outcome to meet the required normality assumption. We performed tests of linkage disequilibrium in unaffected siblings for the SNPs in the final models for the three outcomes using LDSELECT version 1.0 (copyright 2004 by Deborah A. Nickerson, Mark Rieder, Chris Carlson, and Qian Yi, University of Washington, United States) with a threshold *R* ^2^ of 0.80.

Figure S2 summarizes the scheme used to develop models for each outcome. Since the modes of expression of the alleles in the SNPs of interest were not known, we first looked at each SNP using three coding schemes: log-additive, Mendelian dominant, and Mendelian recessive (Step 1). We simplified subsequent analyses by removing from further consideration those SNPs with no significant main effects in any coding scheme (Step 2). Removing those SNPs was a conservative approach, potentially biasing our tests towards the null hypotheses, since the SNPs were prevented from possibly entering the joint action models after adjustment for other variables. We generally coded the remaining SNPs using the schemes that produced the smallest *p* values, since these provided our best estimates of the modes of expression in our data (Step 3). For each outcome, we then created multiple sets of SNPs, where each set contained only SNPs with at least a certain number of non-missing values (Step 4). This was done to address issues due to missing values.

While most SNPs had fairly complete data, others had missing values from substantial numbers of participants: up to 18% of cases and up to 32% of case-sib pairs. We therefore chose an approach where we constructed candidate models using sets of SNPs with fairly complete data (effective sample sizes close to the maximum 443) to explain as much of the outcomes as possible, then checked to see if adding other SNPs on top of the candidate models would contribute significantly. We constructed the candidate models for each set using standard automated procedures (Step 5) and selected a final candidate model for each outcome based on significance and goodness-of-fit (Step 6). We then added other SNPs, which were significant given the candidate models (Step 7) and significant pair-wise interactions (Step 8).

To compare the significance of our axon-guidance pathway SNP models to the significance of randomly selected genomic SNP models, we constructed 4,000 models for each outcome by randomly selecting the appropriate number of SNPs from the entire available dataset. We then plotted the distributions of the test statistics from those models and the values of the test statistics from our final models.

### Validation with a second whole-genome association dataset.

A second whole-genome association study of PD was recently published, including first stage analysis and public release of the data [18]. That study included 276 patients with PD and 276 neurologically normal and unrelated controls. The samples used for that study were derived from the National Institute of Neurological Disorders and Stroke Neurogenetics repository hosted by the Coriell Institute for Medical Research (https://queue.coriell.org/Q/snp_index.asp). There were 408,803 SNPs individually genotyped, and call rates and Hardy-Weinberg equilibrium *p* values were previously reported [23]. We downloaded the individual level data for that study from the Coriell Institute website, and we identified SNPs in that secondary dataset that were assigned to the genes represented by SNPs in each of the predictive genetic models for our primary dataset (same genes and outcomes, different SNPs and samples). We then employed the same statistical methods to construct predictive genetic models for the same outcomes in the secondary dataset, with two exceptions. First, because the secondary dataset employed unrelated controls that were not individually matched, we performed unconditional logistic regression analyses instead of conditional logistic regression analyses for the PD susceptibility outcome. Second, we restricted the age at onset of PD analyses in the secondary dataset to exclude participants with ages younger than those reported in the primary dataset, which resulted in the exclusion of one participant whose age at onset was reportedly 13 years.

### Validation with a gene-expression profiling dataset.

We explored an available gene-expression profiling dataset to determine if there was convergence of those functional data with the genetic association data and models [19]. That study included multiregional gene expression data from postmortem brain specimens from 22 PD cases and 23 normal aged brain donors and represents the most comprehensive expression profiling study of PD to date (largest numbers of participants, brain regions studied, and genes assayed) [56]. Very strict RNA quality control criteria were used. We analyzed data derived from Affymetrix Human Genome U133 Plus 2.0 GeneChip arrays, which included probe set data for 126 of the 128 brain-expressed axon-guidance pathway genes that we initially identified (see Bioinformatic methods). We analyzed probe set data for the substantia nigra, putamen, and caudate regions using methods and criteria similar to the published study [19]. Supporting information regarding the study design, expression values calculations, and array normalization methods for the published study are also provided in Text S1.

For the differential expression analyses of this study, we identified probe sets from within the original dataset that were assigned to the axon-guidance pathway genes of interest (those represented by SNPs in either of the three predictive genetic models in the primary whole-genome association dataset). For each probe set we compared expression for cases and controls in each of the three nigrostriatal regions (Table S6). We considered a probe set informative in a given region if it was expressed in at least 75% of cases or 75% of controls. Although differential expression of a single probe set is most of the times enough to characterize a gene as differentially expressed (multiple polyadenylation sites are represented on Affymetrix gene chips to account for multiple gene transcripts), for this study we employed a more conservative definition of differential gene expression than for the original study [19]. We defined a gene as differentially expressed in a given region if at least one accurate-type (at) probe set assigned to the gene had a *t*-test *p* value <0.05 and absolute value of the fold expression ≥1.3. Alternatively, we defined a gene as differentially expressed in a given region if at least 30% of accurate-type (at) or cross reacting-type (x_at, s_at) probe sets assigned to the gene had *t*-test *p* values <0.05 and absolute values of the fold expression ≥1.3. These definitions weigh the significance of cross-reactive type probe sets lower than accurate-type probe sets because they have less specificity, and also account for the possibility that multiple probe sets for the same gene may not provide concordant gene expression measurements [57].

We also performed a sensitivity analysis where a gene was defined as differentially expressed in a given region if at least one probe set of any type assigned to the gene had a *t*-test *p* value <0.05 and absolute value of the fold expression ≥1.3. This was consistent with the differential expression analyses performed for the original study [19]. Finally, we performed a second sensitivity analysis whereby we employed the same CEL file data as for the original gene expression profiling study [18], and we renormalized and reanalyzed the data using a second standard software package (GeneSpring, Agilent Technologies, http://www.home.agilent.com). That normalization procedure allowed us to obtain informative results for all probe sets and in all 45 genes. Additional details of this sensitivity analysis are provided as supporting information (Text S1).

For our primary differential expression analyses, we tested for possible bias due to the number of informative probe sets per gene by comparing the distributions for differentially expressed versus normally expressed genes using the Wilcoxon rank sum test. For the primary and two sensitivity analyses, we coded the differential expression of genes as increased or reduced if all probe sets assigned to the gene had the same direction of effect or ambiguous if the probe sets had opposite directions of effect.

## Supporting Information

Figure S1 Distributions of the Test Statistics from the Models with Randomly Selected SNPs and the Values of the Test Statistics Figure S1 shows results from our final models for PD susceptibility (A), survival free of PD (B), and age at onset of PD (C), within our primary whole-genome association dataset [11].
(19 KB PDF)

Figure S2 Summary of the Scheme Used to Develop Models for Each Outcome The procedure employed to build joint action models using SNPs from genes in the axon guidance pathway that predict PD susceptibility, survival free of PD, and age at onset of PD, within both whole-genome association datasets [11,18] is presented.
(16 KB PDF)

Figure S3 Goodness-of-Fit of Final Model Using Axon Guidance Genes to Predict Susceptibility to PD Model is within a second whole-genome association dataset [18]. Histogram of predicted probabilities of PD in cases (A) and histogram of predicted probabilities of PD in unrelated controls (B) are presented. A perfect fit would have all predicted probabilities for cases equal to one, and all predicted probabilities for unrelated controls equal to zero. A model with no explanatory value would have histograms that were indistinguishable from each other.
(12 KB PDF)

Figure S4 Kaplan-Meier Survival Plot for Age at Onset in PD Patients Data are grouped by categorized risk score from proportional hazards model, within a second whole-genome association dataset [18]. The model clearly differentiates between early-age-at-onset cases and late-age-at-onset cases. Cases generating the long-dashed line were predicted to be at highest risk for early onset of PD, followed by the medium-dashed, short-dashed, and continuous lines respectively. Since only cases were included, all of the lines end at 0% free of PD.
(10 KB PDF)

Figure S5 Predicted Versus Reported Age at Onset in PD Patients Data presented are within a second whole-genome association dataset [18]. Points indicate reported individual values, and the line represents perfect agreement. In this case, the pattern shows a fairly tight elliptical pattern, reflecting a good fit and high *R* ^2^ of 0.71. The fit appears equally good from the minimum to maximum reported age at onset. By contrast to our primary dataset, which reported age at onset to the month, the second dataset reported age at onset to the year. This and smaller sample size may account for the slightly grainy appearance of the plot for this secondary study as compared to the primary study.
(23 KB PDF)

Table S1 Detailed SNP Identifier Information and Additional Results for Three Outcomes For each of the 1,460 axon-guidance pathway SNPs included in our primary whole-genome association dataset [11], provided are detailed SNP identifier information and additional results for three outcomes: PD susceptibility, survival free of PD, and predicted age at onset of PD. The individual level data for the original whole-genome association study [11] can be downloaded from the NCBI website (http://www.ncbi.nlm.nih.gov/projects/gap/cgi-bin/study.cgi?id=phs000048, anticipated availability within two months of publication).
(3.7 MB XLS)

Table S2 Detailed SNP Identifier Information for the 45 Axon-Guidance Pathway Genes For the secondary whole-genome association dataset [18], provided are detailed SNP identifier information for the 45 axon-guidance pathway genes highlighted by the primary study and additional results for three outcomes: PD susceptibility, survival free of PD, and predicted age at onset of PD.
(1.6 MB XLS)

Table S3 SNPs in Axon-Guidance Pathway Genes Predicting PD Susceptibility Data presented are within a second whole-genome association dataset [18].
(84 KB DOC)

Table S4 SNPs in Axon-Guidance Pathway Genes Predicting Survival Free of PD Data presented are within a second whole-genome association dataset [18].
(71 KB DOC)

Table S5 SNPs in Axon-Guidance Pathway Genes Predicting Age at Onset of PD Data presented are within a second whole-genome association dataset [18].
(77 KB DOC)

Table S6 Detailed Gene Expression Data for Each of the Three Nigrostriatal Regions Considered For the 45 axon-guidance pathway genes represented by SNPs in the three predictive genetic models (as defined by our primary whole-genome association dataset [11]), provided are detailed gene expression data for each of the three nigrostriatal regions considered, including probe set type, *t*-test *p* value, fold expression difference in cases and controls, and percent of cases and controls with expression present for the probe set. The CEL file data for the substantia nigra from the original gene expression profiling study [19] can now be downloaded from the Gene Expression Omnibus website (http://www.ncbi.nlm.nih.gov/geo/query/acc.cgi?acc=GSE7621).
(47 KB XLS)

Text S1 Study Design, Expression Values Calculation, and Array Normalization Document provides additional details regarding the methods employed for the gene expression profiling study [19], including study design, expression values calculation, and array normalization; additional details regarding the differential expression analyses for this study are presented.
(33 KB DOC)

Text S2 MIAME Guidelines Checklist (76 KB DOC)

### Accession Numbers

The National Center for Biotechnology Information Entrez Gene website (http://www.ncbi.nlm.nih.gov/entrez/query.fcgi?db=gene) accession numbers (GeneIDs) for the genes named in the paper include: *ABL1* (25), *ABLIM2* (84448), *CDC42* (998), *CHP* (11261), *CXCR4* (7852), *DCC* (1630), *DPYSL2* (1808), *EFNA5* (1946), *EPHA4* (2043), *EPHA8* (2046), *EPHB1* (2047), *EPHB2* (2048), *FYN* (2534), *GNAI3* (2773), *GSK3B* (2932), *KRAS* (3845), *MRAS* (22808), *NFATC2* (4773), *NFATC4* (4776), *NTNG1* (22854), *PAK1* (5058), *PAK3* (5063), *PAK4* (10298), *PAK6* (56924), *PAK7* (57144), *PLXNA2* (5362), *PLXNC1* (10154), *PPP3CA* (5530), *RAC2* (5880), *ROBO1* (6091), *ROBO2* (6092), *ROCK2* (9475), *RRAS2* (22800), *SEMA3A* (10371), *SEMA3D* (223117), *SEMA3E* (9723), *SEMA4D* (10507), *SEMA5A* (9037), *SLIT2* (9353), *SLIT3* (6586), *SRGAP1* (57522), *SRGAP3* (9901), *UNC5A* (90249), *UNC5C* (8633), and *UNC5D* (137970).

## Abbreviations

CI: confidence interval
HR: hazards ratio
KEGG: Kyoto Encyclopedia of Genes and Genomes
OR: odds ratio
PD: Parkinson disease
SNP: single nucleotide polymorphism
