# Supplementary material for: A Genomic Pathway Approach to a Complex Disease: Axon Guidance and Parkinson Disease
Source: PLoS Genet. 2007 Jun 15;3(6):e98. doi: 10.1371/journal.pgen.0030098 (PMC1904362; doi:10.1371/journal.pgen.0030098)
Supplement: Supplementary file 1 [file pgen.0030098.sg001.pdf]

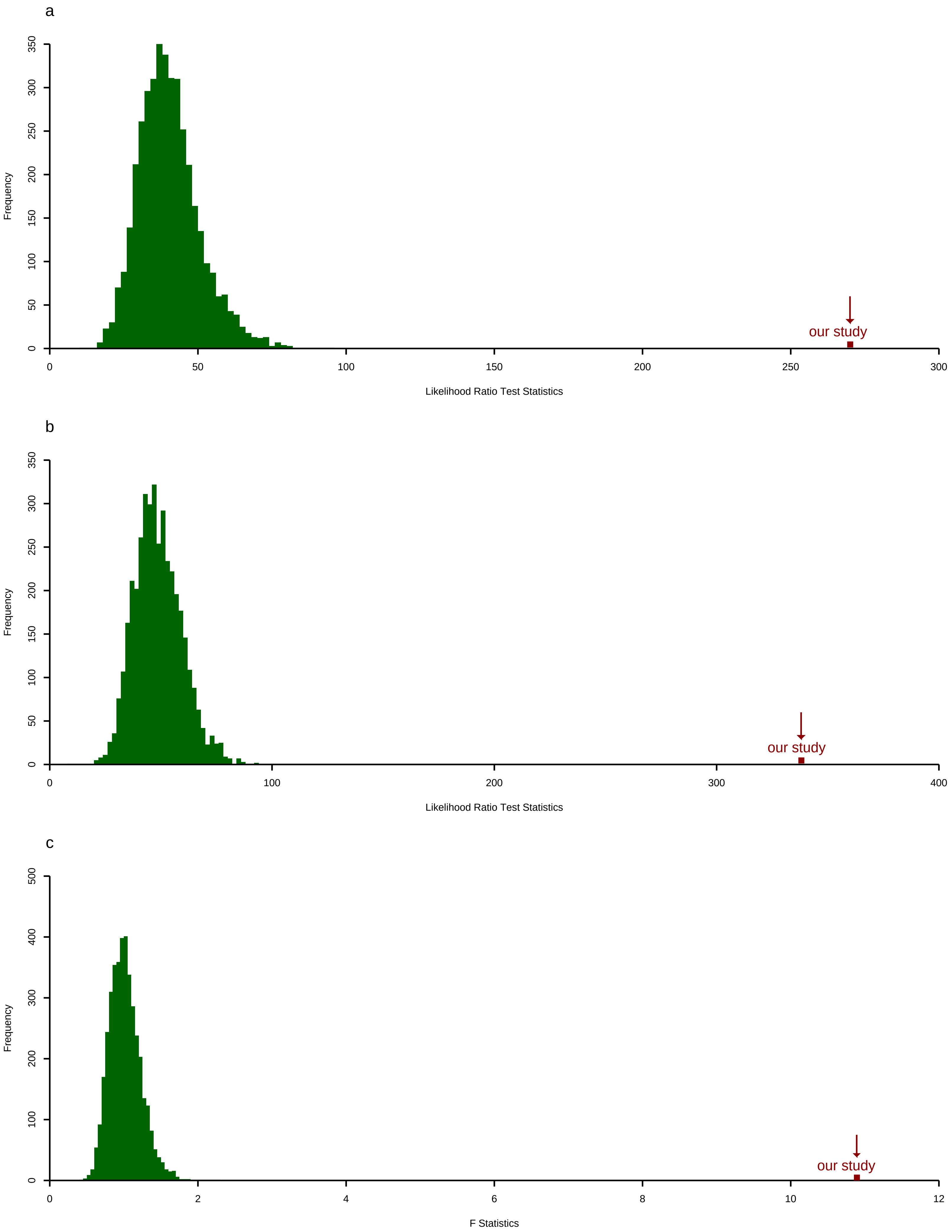
a
Frequency
0
50
100
150
200
250
300
350
our study
0
50
100
150
200
250
300
Likelihood Ratio Test Statistics
b
Frequency
0
50
100
150
200
250
300
350
our study
0
100
200
300
400
Likelihood Ratio Test Statistics
c
Frequency
0
100
200
300
400
500
our study
0
2
4
6
8
10
12
F Statistics
