# Supplementary material for: A Genomic Pathway Approach to a Complex Disease: Axon Guidance and Parkinson Disease
Source: PLoS Genet. 2007 Jun 15;3(6):e98. doi: 10.1371/journal.pgen.0030098 (PMC1904362; doi:10.1371/journal.pgen.0030098)
Supplement: Supplementary file 2 [file pgen.0030098.sg002.pdf]

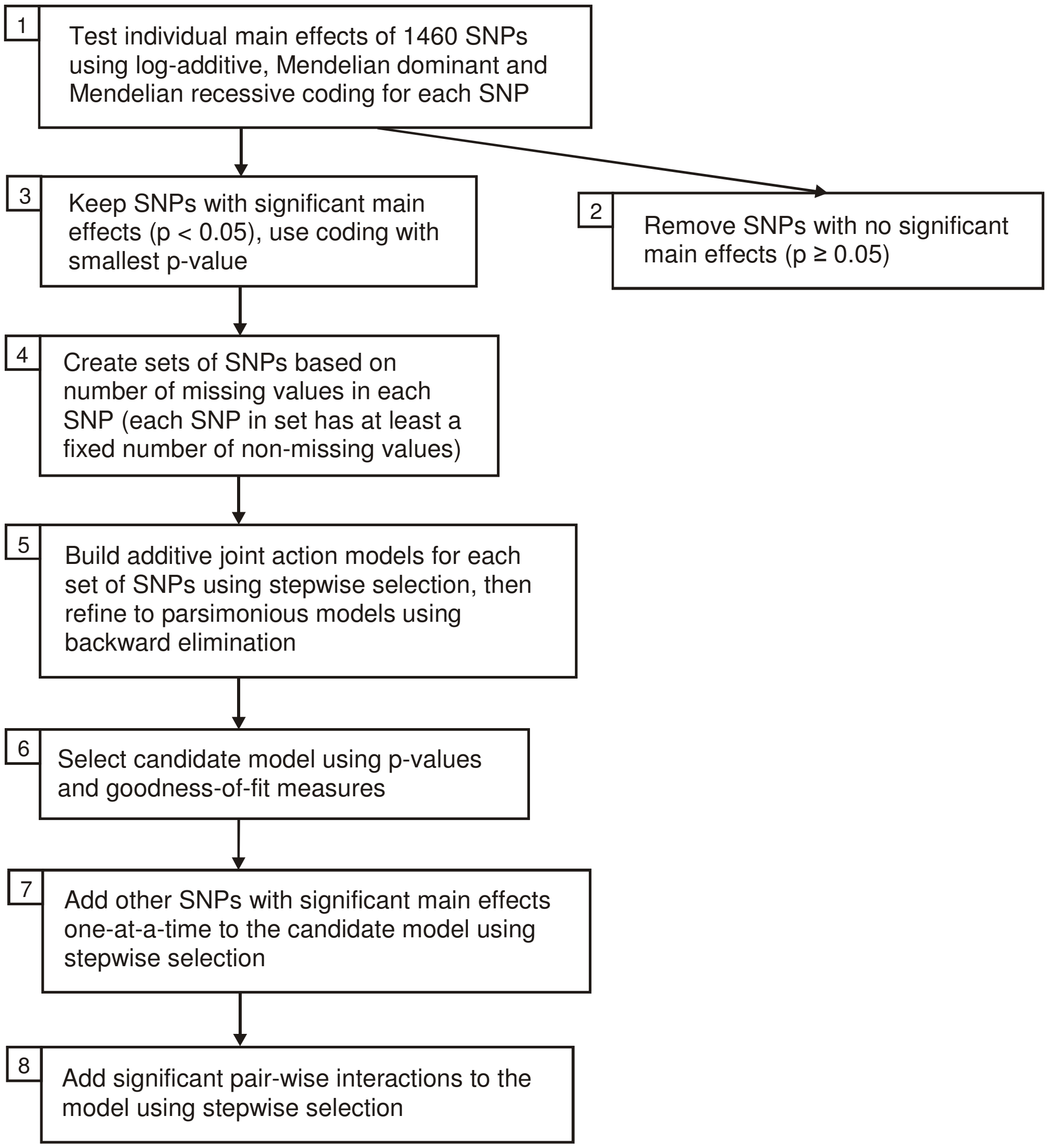
1
Test individual main effects of 1460 SNPs using log-additive, Mendelian dominant and Mendelian recessive coding for each SNP
2
Remove SNPs with no significant main effects (p ≥ 0.05)
3
Keep SNPs with significant main effects (p < 0.05), use coding with smallest p-value
4
Create sets of SNPs based on number of missing values in each SNP (each SNP in set has at least a fixed number of non-missing values)
5
Build additive joint action models for each set of SNPs using stepwise selection, then refine to parsimonious models using backward elimination
6
Select candidate model using p-values and goodness-of-fit measures
7
Add other SNPs with significant main effects one-at-a-time to the candidate model using stepwise selection
8
Add significant pair-wise interactions to the model using stepwise selection
