# Supplementary material for: A Genomic Pathway Approach to a Complex Disease: Axon Guidance and Parkinson Disease
Source: PLoS Genet. 2007 Jun 15;3(6):e98. doi: 10.1371/journal.pgen.0030098 (PMC1904362; doi:10.1371/journal.pgen.0030098)
Supplement: Supplementary file 3 [file pgen.0030098.sg003.pdf]

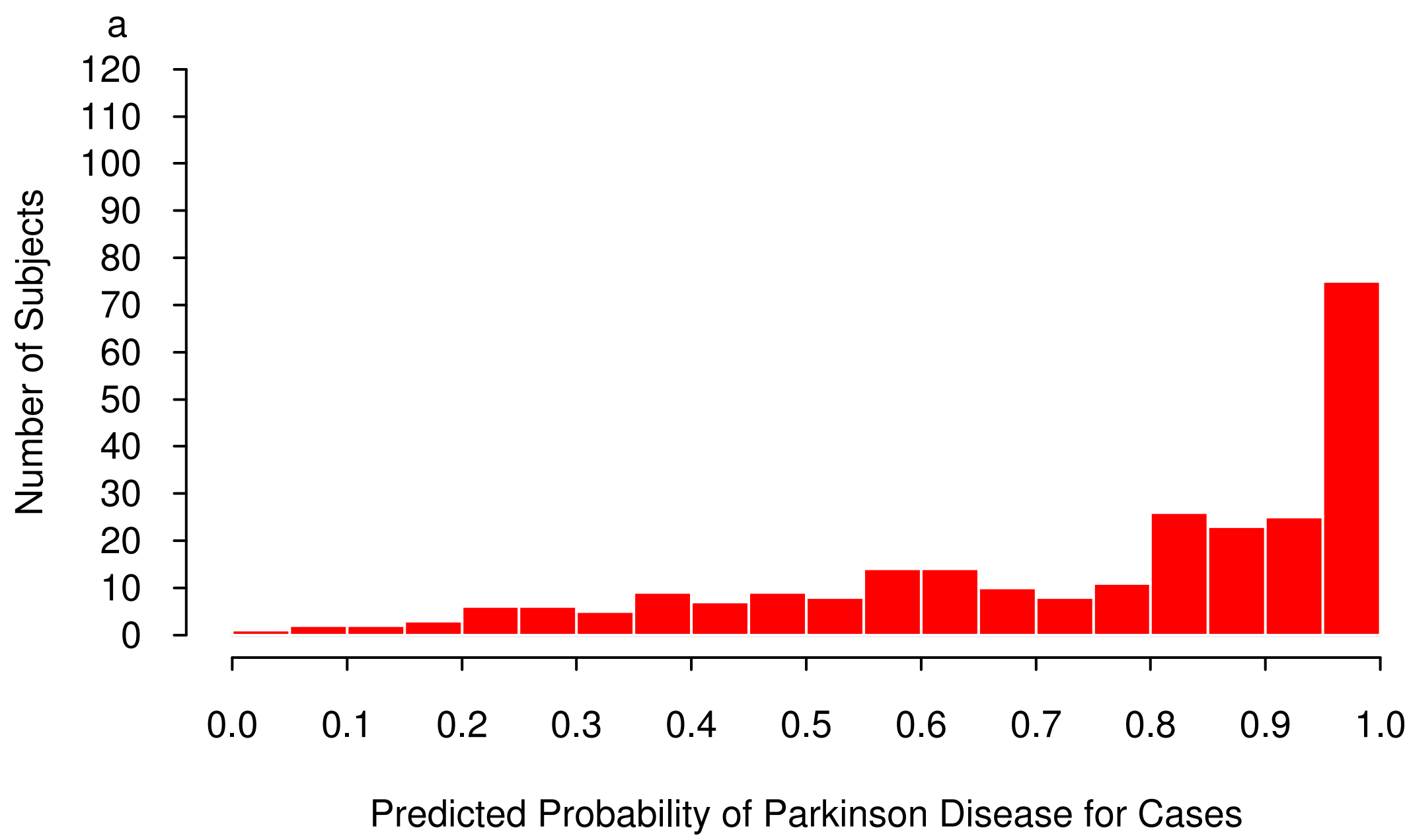
a
120
110
100
90
80
70
60
50
40
30
20
10
0
Number of Subjects
0.0
0.1
0.2
0.3
0.4
0.5
0.6
0.7
0.8
0.9
1.0
Predicted Probability of Parkinson Disease for Cases

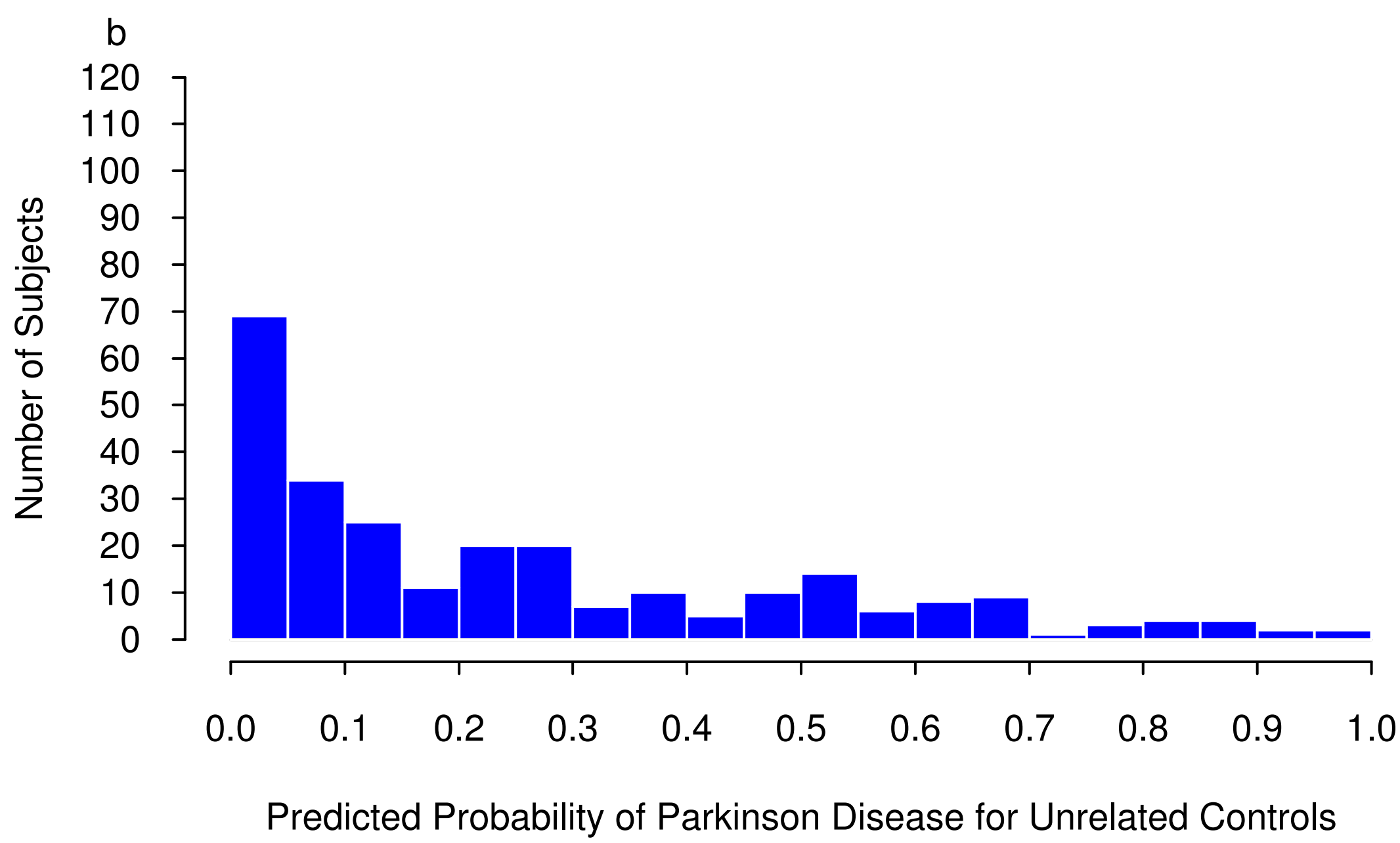
b
120
110
100
90
80
70
60
50
40
30
20
10
0
Number of Subjects
0.0
0.1
0.2
0.3
0.4
0.5
0.6
0.7
0.8
0.9
1.0
Predicted Probability of Parkinson Disease for Unrelated Controls
