# Supplementary material for: A Genomic Pathway Approach to a Complex Disease: Axon Guidance and Parkinson Disease
Source: PLoS Genet. 2007 Jun 15;3(6):e98. doi: 10.1371/journal.pgen.0030098 (PMC1904362; doi:10.1371/journal.pgen.0030098)
Supplement: Supplementary file 4 [file pgen.0030098.sg004.pdf]

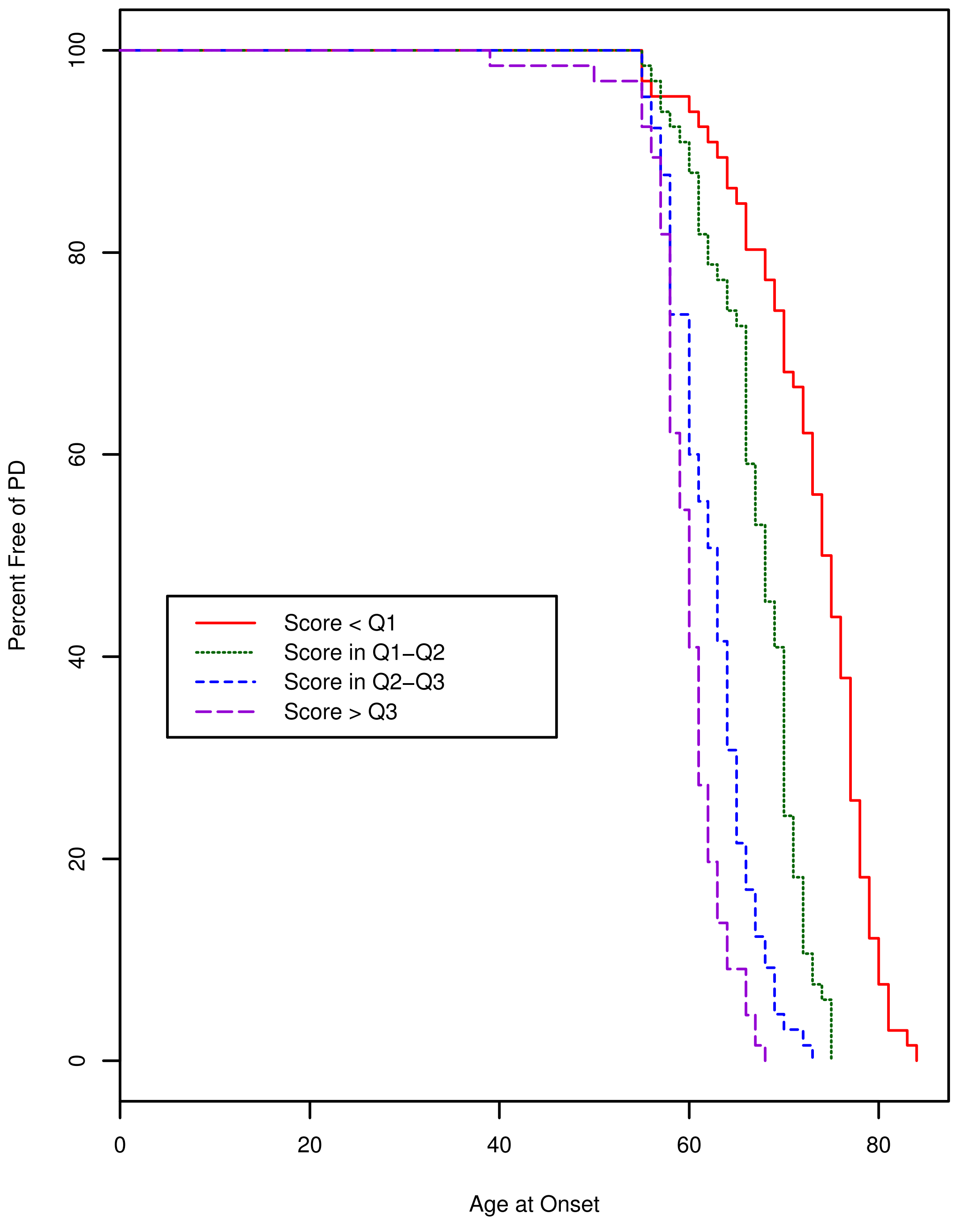

Percent Free of PD
Age at Onset
0
20
40
60
80
100
0
20
40
60
80
Score < Q1
Score in Q1–Q2
Score in Q2–Q3
Score > Q3
