# Supplementary material for: A Genomic Pathway Approach to a Complex Disease: Axon Guidance and Parkinson Disease
Source: PLoS Genet. 2007 Jun 15;3(6):e98. doi: 10.1371/journal.pgen.0030098 (PMC1904362; doi:10.1371/journal.pgen.0030098)
Supplement: Supplementary file 5 [file pgen.0030098.sg005.pdf]

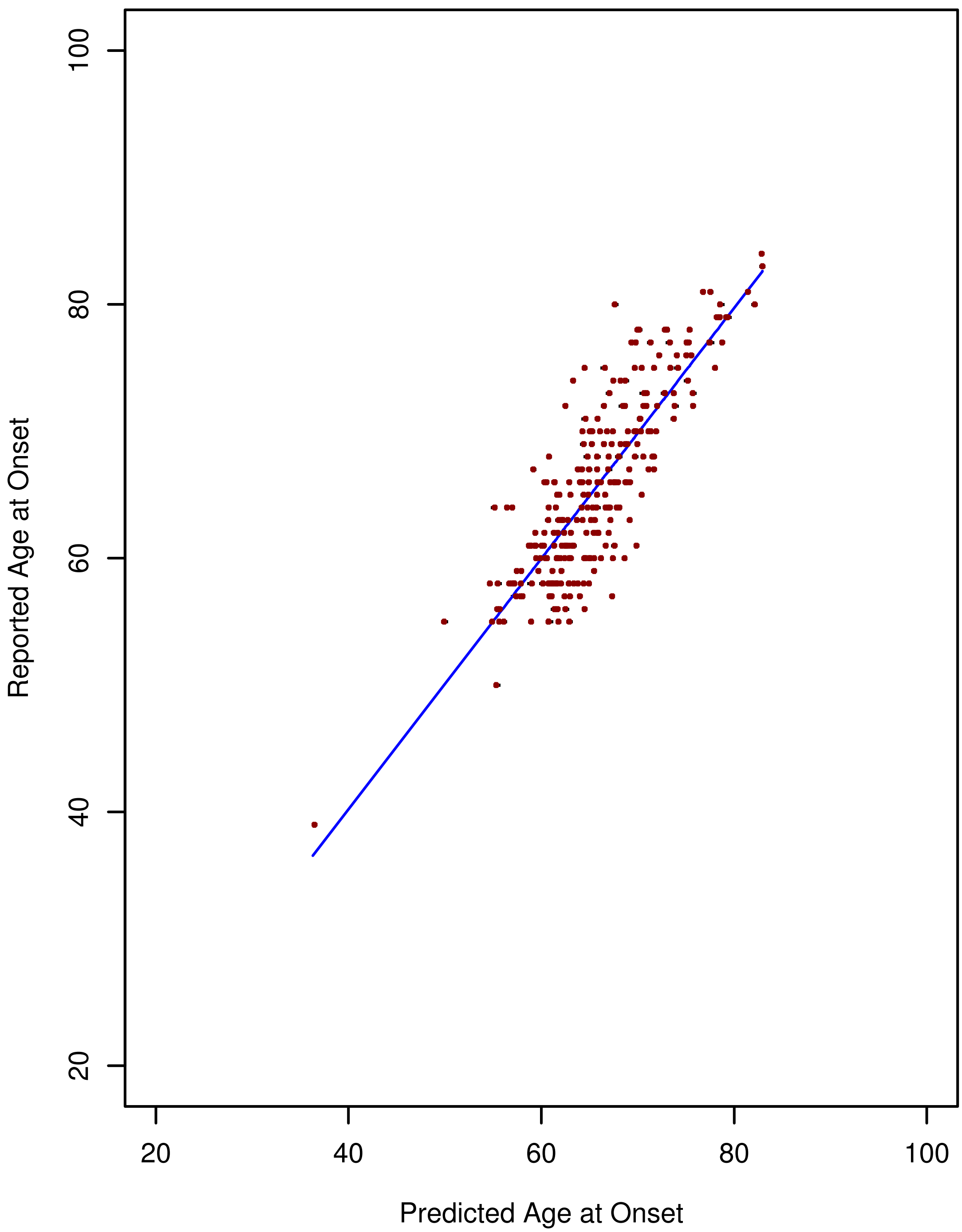
Reported Age at Onset
100
80
60
40
20
20
40
60
80
100
Predicted Age at Onset
