# Supplementary material for: A Genomic Pathway Approach to a Complex Disease: Axon Guidance and Parkinson Disease
Source: PLoS Genet. 2007 Jun 15;3(6):e98. doi: 10.1371/journal.pgen.0030098 (PMC1904362; doi:10.1371/journal.pgen.0030098)
Supplement: Supplementary file 6 [file pgen.0030098.sd001.doc]

**Text S1.** Word document providing additional details regarding the methods employed for the published gene expression profiling study, including study design, expression values and calculation, and array normalization; additional details regarding the differential expression analyses for this study.

Postmortem brain tissue was obtained from University of Miami/NPF Brain Endowment Bank donors diagnosed with neuropathologically confirmed PD [1] (n=22) or aged individuals with no history or pathological diagnosis of neurologic or psychiatric disease (n=23). Detailed demographics of our cohort can be found in [2]. All patients were carefully characterized for clinical phenotype, treatment, and agonal state. Regional samples of postmortem brain were taken from frozen coronal blocks based on surface and cytoarchitectural landmarks after controlling for important determinants of RNA quality (postmortem interval, brain pH).

Whole tissue from the substantia nigra, putamen, and caudate was used to conduct gene microarray experiments. Total RNA isolation was performed using a TriZol method and RNeasy columns, and labeled cRNA was prepared according to the manufacturer’s protocol (Affymetrix, Santa Clara, CA). Total RNA from each sample was used to prepare biotinylated target RNA, following the manufacturers’ recommendations (<http://www.affymetrix.com/support/technical/manual/expression_manual.affx>). The target cDNA generated from each sample was processed as per manufacturer's recommendation using an Affymetrix GeneChip Instrument System (<http://www.affymetrix.com/support/technical/manual/expression_manual.affx>). The samples were checked for evidence of degradation and integrity, A260/A280>1.9 and 28S/18S>1.6, (2100 Bioanalyzer; Agilent Technologies, Palo Alto, Calif ). The Human Genome U133 Plus 2.0 GeneChips were used (information available at: <http://www.affymetrix.com/products/arrays/specific/hgu133plus.affx>). Microarray quality control parameters included the following: background, noise (RawQ), consistent number of genes detected as present across arrays, consistent scale factors, and consistent β-actin and glyceraldehyde-3-phosphate dehydrogenase 5’/3’ signal ratios.

We analyzed the gene expression from three different brain regions in PD patients and their age matched normal subjects. After checking for microarray quality control the following number of samples were used in the analysis of genes in the constructed models of axonal guidance: for substantia nigra 16 Parkinson’s disease patients vs 8 controls, for putamen 13 Parkinson’s disease patients vs 7 controls, and for caudate nucleus 14 Parkinson’s disease patients vs 9 controls (22 cases and 23 controls total).

We selected genes on the basis of “present calls” by Microarray Analysis Suite 5.0. For a gene to be included, it had to be present (detectable) in at least 75% of the subjects in at least 1 of the 2 groups (Parkinson’s disease patients and controls) to reduce the chances of false-positive findings. Expression data were analyzed using Genesis (GeneLogic, Gaithersburg, MD) and AVADIS software (Strand Genomics, Redwood City, CA ). Following normalization one-way analysis of variance was performed for each gene to identify statistically significant gene expression changes. Two criteria were used to determine whether a gene was differentially expressed: p value ≤ .05 and fold-change (FC) of ± 1.3.

For this study we performed additional differential expression analyses (sensitivity analyses). Gene chip raw Affymetrix data in the form of CEL files were also uploaded in GeneSpring v7.2 (Agilent Technologies), normalized with GC-RMA, and re-analyzed using the same statistical tests and criteria.

**References**

1.	Hughes AJ, Daniel SE, Kilford L, Lees AJ. (1992) Accuracy of clinical diagnosis of idiopathic Parkinson's disease: a clinico-pathological study of 100 cases. J Neurol Neurosurg Psychiatry 1992;55(3):181-184.

2.	Papapetropoulos S, Ffrench-Mullen J, McCorquodale D, Qin Y, Pablo J, Mash DC. (2006) Multiregional gene expression profiling identifies MRPS6 as a possible candidate gene for Parkinson's disease. Gene Expr 2006;13(3):205-215.
