# Supplementary material for: A Genomic Pathway Approach to a Complex Disease: Axon Guidance and Parkinson Disease
Source: PLoS Genet. 2007 Jun 15;3(6):e98. doi: 10.1371/journal.pgen.0030098 (PMC1904362; doi:10.1371/journal.pgen.0030098)
Supplement: Supplementary file 7 [file pgen.0030098.sd002.doc]

# MIAME Checklist

Lesnick TG, Papapetropoulos S, Mash DC, Ffrench-Mullen J, Shehadeh L, et al. (2007) A genomic pathway approach to a complex disease: Axon guidance and Parkinson disease. PLoS Genet 3(6): e98. doi:10.1371/journal.pgen.0030098

Experiment Design:

1. *Goal:* To employ an available gene expression profiling dataset [1] to externally validate predictive genetic models of Parkinson’s disease that included SNPs from 45 axon guidance pathway genes.
2. *Brief description:* We explored an available gene expression profiling dataset to determine if there was convergence of functional data from microarray experiments [1] with the genetic association data and models. The gene expression profiling data were derived from postmortem brain specimens of Parkinson’s disease cases and normal aged brain donors. Very strict RNA quality control criteria were used. We analyzed data from Affymetrix Human Genome U133 Plus 2.0 GeneChips®, which included probe set data for the axon guidance pathway genes that we identified. We analyzed probe set data for the substantia nigra, putamen, and caudate regions using methods and criteria similar to the published study [1].
3. *Keywords:* Parkinson’s disease, axon guidance pathway, postmortem brain, genomic convergence
4. *Experimental factors:* gene expression variation
5. *Experimental design:* characteristics of individual patients, relationships between Parkinson’s disease patients and normal controls
6. *Quality control steps taken:*
   a. RNA was checked for evidence of degradation and integrity (2100 Bioanalyzer; Agilent Technologies, Palo Alto, Calif ).
   b. Microarray quality control parameters included the following: background, noise (RawQ), consistent number of genes detected as present across arrays, consistent scale factors, and consistent β-actin and glyceraldehyde-3-phosphate dehydrogenase 5’/3’ signal ratios.
   c. We selected genes on the basis of “present calls” by Microarray Analysis Suite 5.0. For a gene to be included, it had to be present (detectable) in at least 75% of the subjects in at least 1 of the 2 groups (Parkinson’s disease patients and controls) to reduce the chances of false-positive findings.
7. *Links to supplemental websites/database accession numbers:* this data will be available through GEO at: http://www.ncbi.nlm.nih.gov/geo/ (GEO Accession number GSE7621).

Samples used, extract preparation and labeling:

1. *Origin and characteristics of each sample:*

| **Sample name** | **Characteristic** | **Gender** | **Title** |
| --- | --- | --- | --- |
| **SAMPLE 1** | Aged Control | female | substantia nigra normal rep1 |
| **SAMPLE 2** | Aged Control | male | substantia nigra normal rep2 |
| **SAMPLE 3** | Aged Control | female | substantia nigra normal rep3 |
| **SAMPLE 4** | Aged Control | female | substantia nigra normal rep4 |
| **SAMPLE 5** | Aged Control | female | substantia nigra normal rep5 |
| **SAMPLE 6** | Aged Control | female | substantia nigra normal rep6 |
| **SAMPLE 7** | Aged Control | male | substantia nigra normal rep7 |
| **SAMPLE 8** | Aged Control | male | substantia nigra normal rep8 |
| **SAMPLE 9** | Aged Control | male | substantia nigra normal rep9 |
| **SAMPLE 10** | Parkinson's Disease | male | substantia nigra PARKINSON’S DISEASE rep1 |
| **SAMPLE 11** | Parkinson's Disease | male | substantia nigra PARKINSON’S DISEASE rep2 |
| **SAMPLE 12** | Parkinson's Disease | male | substantia nigra PARKINSON’S DISEASE rep3 |
| **SAMPLE 13** | Parkinson's Disease | male | substantia nigra PARKINSON’S DISEASE rep4 |
| **SAMPLE 14** | Parkinson's Disease | male | substantia nigra PARKINSON’S DISEASE rep5 |
| **SAMPLE 15** | Parkinson's Disease | female | substantia nigra PARKINSON’S DISEASE rep6 |
| **SAMPLE 16** | Parkinson's Disease | male | substantia nigra PARKINSON’S DISEASE rep7 |
| **SAMPLE 17** | Parkinson's Disease | male | substantia nigra PARKINSON’S DISEASE rep8 |
| **SAMPLE 18** | Parkinson's Disease | female | substantia nigra PARKINSON’S DISEASE rep9 |
| **SAMPLE 19** | Parkinson's Disease | male | substantia nigra PARKINSON’S DISEASE rep10 |
| **SAMPLE 20** | Parkinson's Disease | male | substantia nigra PARKINSON’S DISEASE rep11 |
| **SAMPLE 21** | Parkinson's Disease | female | substantia nigra PARKINSON’S DISEASE rep12 |
| **SAMPLE 22** | Parkinson's Disease | male | substantia nigra PARKINSON’S DISEASE rep13 |
| **SAMPLE 23** | Parkinson's Disease | male | substantia nigra PARKINSON’S DISEASE rep14 |
| **SAMPLE 24** | Parkinson's Disease | male | substantia nigra PARKINSON’S DISEASE rep15 |
| **SAMPLE 25** | Parkinson's Disease | male | substantia nigra PARKINSON’S DISEASE rep16 |

1. Postmortem brain tissue was obtained from University of Miami/NPF Brain Endowment Bank donors diagnosed with neuropathologically confirmed PD [3]. All patients were carefully characterized for clinical phenotype, treatment, and agonal state. Regional samples of postmortem brain were taken from frozen coronal blocks based on surface and cytoarchitectural landmarks after controlling for important determinants of RNA quality (postmortem interval, brain pH).
2. Whole tissue from the substantia nigra was used to conduct gene microarray experiments. Total RNA isolation was performed using a TriZol method and RNeasy columns, and labeled cRNA was prepared according to the manufacturer’s protocol (Affymetrix, Santa Clara, CA). Total RNA from each sample was used to prepare biotinylated target RNA, following the manufacturers’ recommendations (<http://www.affymetrix.com/support/technical/manual/expression_manual.affx>). The target cDNA generated from each sample was processed as per manufacturer's recommendation using an Affymetrix GeneChip Instrument Syst. (<http://www.affymetrix.com/support/technical/manual/expression_manual.affx>).

Hybridization Conditions and Parameters:

The protocol and conditions used for hybridization, blocking and washing, including any post-processing steps is available at the following url: <http://www.affymetrix.com/support/technical/manuals.affx>

Measurement Data and Specifications:

1. *Data*
   a. Raw data: Standard .CEL files format (<http://www.ncbi.nlm.nih.gov/geo/>) (GEOAccession number GSE7621).
2. *Data extraction and processing protocols*
   a. *Image scanning hardware and software, and processing procedures and parameters:* Microarray hybridization was detected using a Affymetrix GeneChip scanner. Gene chip analysis was performed with Microarray Analysis Suite version 5.0, Data Mining Tool 2.0, and Microarray database software (available at: [http://www.affymetrix.com](http://www.affymetrix.com/)).
   b. *Normalization, transformation and data selection procedures and parameters.* We selected genes on the basis of “present calls” by Microarray Analysis Suite 5.0. For a gene to be included, it had to be present (detectable) in at least 75% of the subjects in at least 1 of the 2 groups (Parkinson’s disease patients and controls) to reduce the chances of false-positive findings. Expression data were analyzed using Genesis (GeneLogic, Gaithersburg, MD) and AVADIS software (Strand Genomics, Redwood City, CA ). Following normalization one-way analysis of variance was performed for each gene to identify statistically significant gene expression changes. Two criteria were used to determine whether a gene was differentially expressed: p value ≤ 0.05 and fold-change (FC) of ± 1.3. Our methods have already been published in [1]. For this validation study we also considered probe set type (i.e. accurate type vs. other), as detailed in the manuscript.

Array Design: Human Genome U133 Plus 2.0 GeneChips were used (information available at: <http://www.affymetrix.com/products/arrays/specific/hgu133plus.affx>). The principal array organism is Homo sapiens.

**References**

1.	Papapetropoulos S, Ffrench-Mullen J, McCorquodale D, Qin Y, Pablo J, Mash DC. Multiregional gene expression profiling identifies MRPS6 as a possible candidate gene for Parkinson's disease. Gene Expr 2006;13(3):205-15.

2.	Fahn S, Elton RL, Members of the UPDRS Development Committee. Unified Parkinson's Disease Rating Scale. In: Fahn S, Marsden CD, Calne DB, editors. Recent developments in Parkinson's disease. 2nd edition ed. Florham Park, NJ: Macmillan Health Care Information; 1987. p. 153-164.

3	Cech, T.R., Sharing Publication-Related Data and Materials: Responsibilities of Authorship in the Life Sciences. (2003). Available at http://darwin.nap.edu/books/0309088593/html

4.	A. Brazma, P Hingamp, J Quackenbush, G Sherlock, P Spellman, C Stoeckert, J Aach, W Ansorge, C A Ball, H C Causton, T Gaasterland, P Glenisson, F C P Holstege, I F Kim, V Markowitz, J C Matese, H Parkinson, A Robinson, U Sarkans, S Schulze-Kremer, J Stewart, R Taylor, J Vilo & M Vingron. (2001). Minimum information about a microarray experiment (MIAME)—toward standards for microarray data, Nature Genetics, 29, 365 - 371. Available at http://www.microarray.be/ng_miame.pdf

5.	C.A. Ball, A. Brazma, H. Causton, S. Chervitz, R. Edgar, P. Hingamp, J.C. Matese, H. Parkinson, J. Quackenbush, M. Ringwald, S. Sansone, G. Sherlock, P. Spellman, C. Stoeckert, Y. Tateno, R. Taylor, J. White and N. Winegarden. (2004). Submission of Microarray Data to Public Repositories. PLoS Biology, 2(9), e317: 1276-1277. Available at http://biology.plosjournals.org/perlserv/?request=get-document&doi=10.1371/journal.pbio.0020317
