# Supplementary material for: A Genomic Pathway Approach to a Complex Disease: Axon Guidance and Parkinson Disease
Source: PLoS Genet. 2007 Jun 15;3(6):e98. doi: 10.1371/journal.pgen.0030098 (PMC1904362; doi:10.1371/journal.pgen.0030098)
Supplement: Supplementary file 8 [file pgen.0030098.st003.doc]

**Table S3** Axon-guidance SNPs predicting Parkinson’s disease susceptibility, within a Secondary Whole-genome Association Dataset [18]

| Gene* (rs ID),  Final Coding Scheme^†^ | Odds Ratio  (95% CI)** | *P* value** | Interaction* | Odds Ratio  (95% CI)** | *P* value** |
| --- | --- | --- | --- | --- | --- |
|  |  |  |  |  |  |
| *CDC42* *(rs2056974)*, d | 2.09 (1.24-3.52) | 5.6x10^-03^ | *DCC_5_* FYN_1_* | 17.01 (3.06-94.59) | 1.2x10^-03^ |
| *DCC_1_* *(rs1160400)*, r | 7.75 (1.06-57.01) | 4.4x10^-02^ | *EPHB1_1_* EPHB1_4_* | 3.82 (1.39-10.48) | 9.3x10^-03^ |
| *DCC_2_* *(rs1466373)*, d | 2.03 (1.14-3.64) | 1.7x10^-02^ | *PLXNC1_1_* PLXNA2_2_* | 29.53 (1.82-478.49) | 1.7x10^-02^ |
| *DCC_3_* *(rs1502219)*, a | 2.42 (1.55-3.79) | 1.0x10^-04^ | *RRAS2* SEMA5A_2_* | 4.85 (1.44-16.33) | 1.1x10^-02^ |
| *DCC_4_* *(rs4334396)*, r | 14.33 (3.92-52.34) | 5.6x10^-05^ | *SLIT3_3_* SEMA5A_2_* | 15.93 (2.00-127.05) | 9.0x10^-03^ |
| *DCC_5_* *(rs7506794)*, a | 0.07 (0.03-0.19) | 1.6x10^-07^ | *RRAS2* EFNA5_3_* | 0.03 (0.001-0.67) | 2.7x10^-02^ |
| *EFNA5_1_* *(rs152580)*, r | 2.78 (1.49-5.19) | 1.3x10^-03^ |  |  |  |
| *EFNA5_2_* *(rs252802)*, d | 2.94 (1.58-5.47) | 7.0x10^-04^ |  |  |  |
| *EFNA5_3_* *(rs6868410)*, r | 0.67 (0.07-7.00) | 7.4x10^-01^ |  |  |  |
| *EPHA4* *(rs10498114)*, d | 0.38 (0.21-0.68) | 1.2x10^-03^ |  |  |  |
| *EPHB1_1_* *(rs11917038)*, a | 0.93 (0.49-1.76) | 8.2x10^-01^ |  |  |  |
| *EPHB1_2_* *(rs1404577)*, r | 0.19 (0.06-0.66) | 8.6x10^-03^ |  |  |  |
| *EPHB1_3_* *(rs6793828)*, r | 0.48 (0.24-0.95) | 3.5x10^-02^ |  |  |  |
| *EPHB1_4_* *(rs9865620)*, d | 0.24 (0.13-0.45) | 6.8x10^-06^ |  |  |  |
| *EPHB2* *(rs11800828)*, d | 1.89 (1.07-3.35) | 3.0x10^-02^ |  |  |  |
| *FYN_1_* *(rs11153311)*, a | 0.36 (0.20-0.64) | 5.5x10^-04^ |  |  |  |
| *FYN_2_* *(rs809193)*, a | 2.31 (1.48-3.62) | 2.4x10^-04^ |  |  |  |
| *GSK3B (rs3108749)*, a | 0.36 (0.20-0.66) | 1.0x10^-03^ |  |  |  |
| *PAK7_1_* *(rs1033470)*, d | 2.58 (1.51-4.43) | 5.7x10^-04^ |  |  |  |
| *PAK7_2_* *(rs219861)*, d | 0.53 (0.30-0.93) | 2.6x10^-02^ |  |  |  |
| *PLXNA2_1_* *(rs2782934)*, r | 0.19 (0.07-0.51) | 1.0x10^-03^ |  |  |  |
| *PLXNA2_2_* *(rs6685511)*, d | 2.26 (1.21-4.22) | 1.1x10^-02^ |  |  |  |
| *PLXNC1_1_* *(rs10507033)*, r | 0.02 (0.003-0.16) | 1.7x10^-04^ |  |  |  |
| *PLXNC1_2_* *(rs865569)*, r | 21.64 (2.41-194.15) | 6.0x10^-03^ |  |  |  |
| *PPP3CA* *(rs2732504)*, d | 0.48 (0.29-0.82) | 6.3x10^-03^ |  |  |  |
| *RAC2* *(rs9610680)*, a | 1.95 (1.19-3.21) | 8.2x10^-03^ |  |  |  |
| *RRAS2* *(rs2970334)*, d | 0.32 (0.17-0.60) | 4.2x10^-04^ |  |  |  |
| *SEMA5A_1_* *(rs1512508)*, d | 2.38 (1.27-4.47) | 6.7x10^-03^ |  |  |  |
| *SEMA5A_2_* *(rs40701)*, r | 0.98 (0.39-2.47) | 9.6x10^-01^ |  |  |  |
| *SLIT3_1_* *(rs11746295)*, r | 0.14 (0.03-0.58) | 6.7x10^-03^ |  |  |  |
| *SLIT3_2_* *(rs1979923)*, r | 0.20 (0.08-0.53) | 1.2x10^-03^ |  |  |  |
| *SLIT3_3_* *(rs884787)*, d | 3.48 (1.44-8.43) | 5.8x10^-03^ |  |  |  |
| *UNC5C* *(rs6820369)*, d | 2.22 (1.03-4.76) | 4.1x10^-02^ |  |  |  |
|  |  |  |  |  |  |

* Subscript is used to identify individual SNPs in interactions.

** Results adjusted for age and gender.

^†^ a = log-additive, d = Mendelian dominant, r = Mendelian recessive.
