# Supplementary material for: A Genomic Pathway Approach to a Complex Disease: Axon Guidance and Parkinson Disease
Source: PLoS Genet. 2007 Jun 15;3(6):e98. doi: 10.1371/journal.pgen.0030098 (PMC1904362; doi:10.1371/journal.pgen.0030098)
Supplement: Supplementary file 9 [file pgen.0030098.st004.doc]

**Table S4** Axon-guidance SNPs predicting survival free of Parkinson’s disease, within a Secondary Whole-genome Association Dataset [18]

| Gene* (rs ID),  Final Coding Scheme^†^ | Hazard Ratio  (95% CI) | *P* value | Interaction* | Hazard Ratio  (95% CI) | *P* value |
| --- | --- | --- | --- | --- | --- |
|  |  |  |  |  |  |
| *DCC_1_* *(rs1675225)*, a | 1.54 (1.12-2.13) | 8.70x10^-03^ | *NTNG1_2_* ROBO1_1_* | 0.25 (0.12-0.51) | 1.70x10^-04^ |
| *DCC_2_* *(rs12456286)*, r | 5.76 (2.16-15.37) | 4.70x10^-04^ |  |  |  |
| *DPYSL2_1_* *(rs327216)*, a | 1.68 (1.16-2.44) | 6.00x10^-03^ |  |  |  |
| *DPYSL2_2_* *(rs2585459)*, r | 0.60 (0.39-0.92) | 2.10x10^-02^ |  |  |  |
| *EPHB1_1_* *(rs868909)*, d | 0.58 (0.44-0.78) | 2.30x10^-04^ |  |  |  |
| *EPHB1_2_* *(rs959562)*, d | 0.50 (0.33-0.74) | 5.50x10^-04^ |  |  |  |
| *EPHB1_3_* *(rs6802089)*, a | 1.28 (1.04-1.59) | 2.20x10^-02^ |  |  |  |
| *EPHB1_4_ (rs10512945)*, a | 1.59 (1.21-2.10) | 1.00x10^-03^ |  |  |  |
| *NFATC2* *(rs4811184)*, r | 4.59 (2.26-9.35) | 2.60x10^-05^ |  |  |  |
| *NTNG1_1_* *(rs3813219)*, r | 0.26 (0.11-0.60) | 1.50x10^-03^ |  |  |  |
| *NTNG1_2_* *(rs12133394)*, d | 0.75 (0.52-1.10) | 1.40x10^-01^ |  |  |  |
| *PLXNC1_1_* *(rs6538491)*, d | 0.55 (0.40-0.77) | 3.40x10^-04^ |  |  |  |
| *PLXNC1_2_* *(rs10507031)*, a | 0.73 (0.60-0.90) | 2.30x10^-03^ |  |  |  |
| *PLXNC1_3_* *(rs12579612)*, r | 2.83 (1.38-5.82) | 4.50x10^-03^ |  |  |  |
| *PPP3CA* *(rs2732504)*, a | 0.75 (0.62-0.92) | 5.10x10^-03^ |  |  |  |
| *ROBO1_1_ (rs3773236)*, d | 2.42 (1.73-3.39) | 2.20x10^-07^ |  |  |  |
| *ROBO1_2_* *(rs12486635)*, r | 1.72 (1.07-2.75) | 2.40x10^-02^ |  |  |  |
| *ROBO2_1_* *(rs2875436)*, r | 3.48 (1.19-10.20) | 2.30x10^-02^ |  |  |  |
| *ROBO2_2_* *(rs7614439)*, d | 1.45 (1.10-1.91) | 7.60x10^-03^ |  |  |  |
| *ROBO2_3_* *(rs7640201)*, d | 4.65 (2.35-9.20) | 9.90x10^-06^ |  |  |  |
| *ROCK2* *(rs726843)*, r | 0.61 (0.45-0.84) | 2.10x10^-03^ |  |  |  |
| *SEMA3E (rs2713178)*, a | 1.43 (1.17-1.76) | 5.80x10^-04^ |  |  |  |
| *SEMA5A* *(rs1805958)*, r | 2.64 (1.31-5.32) | 6.70x10^-03^ |  |  |  |
| *SLIT3_1_* *(rs11134568)*, r | 0.30 (0.13-0.70) | 5.70x10^-03^ |  |  |  |
| *SLIT3_2_* *(rs11954621)*, d | 0.71 (0.53-0.95) | 2.30x10^-02^ |  |  |  |
| *SRAGP3_1_* *(rs370526)*, d | 0.76 (0.58-0.99) | 4.20x10^-02^ |  |  |  |
| *SRGAP3_2_* *(rs557377)*, r | 0.22 (0.12-0.39) | 2.70x10^-07^ |  |  |  |
| *SRGAP3_3_* *(rs1043951)*, a | 1.72 (1.28-2.32) | 3.80x10^-04^ |  |  |  |
| *SRGAP3_4_* *(rs3732977)*, r | 0.10 (0.03-0.32) | 1.10x10^-04^ |  |  |  |
| *SRGAP3_5_* *(rs6793348)*, d | 0.63 (0.47-0.84) | 1.30x10^-03^ |  |  |  |
| *SRGAP3_6_* *(rs17530358)*, r | 2.28 (1.41-3.69) | 8.30x10^-04^ |  |  |  |
|  |  |  |  |  |  |

* Subscript is used to identify individual SNPs in interactions.

^†^ a = log-additive, d = Mendelian dominant, r = Mendelian recessive.
