# Supplementary material for: A Genomic Pathway Approach to a Complex Disease: Axon Guidance and Parkinson Disease
Source: PLoS Genet. 2007 Jun 15;3(6):e98. doi: 10.1371/journal.pgen.0030098 (PMC1904362; doi:10.1371/journal.pgen.0030098)
Supplement: Supplementary file 10 [file pgen.0030098.st005.doc]

**Table S5** Axon-guidance SNPs predicting age at onset of Parkinson’s disease, within a Secondary Whole-Genome Association Dataset [18]

| Gene* (rs ID),  Final Coding Scheme^†^ | Regression coefficient  (standard error) | *P* value | Interaction* | Regression coefficient  (standard error) | *P* value |
| --- | --- | --- | --- | --- | --- |
|  |  |  |  |  |  |
| (Intercept) | 3795.40 (147.38) | 4.3x10^-74^ | *NTNG1_2_* NTNG1_1_* | 1012.50 (491.71) | 4.1x10^-02^ |
| *ABLIM2 (rs1574393)*, d | -49.84 (113.59) | 6.6x10^-01^ | *PAK3* NTNG1_1_* | -1400.09 (635.70) | 2.9x10^-02^ |
| *DCC_1_* *(rs11082952)*, d | 204.18 ( 85.79) | 1.8x10^-02^ | *DCC_1_* DPYSL2* | 616.39 (253.12) | 1.6x10^-02^ |
| *DCC_2_* *(rs6508153)*, d | 494.94 ( 75.56) | 3.0x10^-10^ | *DPYSL2* ABLIM2* | 1081.60 (269.80) | 8.1x10^-05^ |
| *DPYSL2* *(rs2585459)*, r | 119.14 (173.95) | 4.9x10^-01^ | *EPHB1_3_* SEMA5A* | -525.25 (232.19) | 2.5x10^-02^ |
| *EFNA5* *(rs252991)*, d | 271.73 ( 78.45) | 6.2x10^-04^ | *EPHB1_1_* SEMA3E_2_* | -849.80 (393.19) | 3.2x10^-02^ |
| *EPHB1_1_ (rs12488683)*, r | -375.90 (140.38) | 7.9x10^-03^ | *EPHB1_1_* SEMA3E_1_* | 1264.51 (467.74) | 7.4x10^-03^ |
| *EPHB1_2_* *(rs12489360)*, a | -233.83 ( 58.97) | 9.4x10^-05^ | *SEMA3E_2_* ABLIM2* | 546.98 (240.42) | 2.4x10^-02^ |
| *EPHB1_3_ (rs2030737)*, a | 140.67 ( 56.03) | 1.3x10^-02^ | *SEMA3E_2_* ROBO1_1_* | 1190.80 (370.50) | 1.5x10^-03^ |
| *EPHB1_4_* *(rs6439552)*, d | 514.37 (139.71) | 2.8x10^-04^ | *SLIT2_1_* EPHB1_4_* | -682.99 (220.23) | 2.2x10^-03^ |
| *NFATC2* *(rs4811184)*, r | -671.00 (190.98) | 5.2x10^-04^ | *SLIT3_2_* ROBO1_1_* | 1629.19 (500.80) | 1.3x10^-03^ |
| *NTNG1_1_* *(rs11803905)*, r | 343.55 (142.64) | 1.7x10^-02^ |  |  |  |
| *NTNG1_2_* *(rs3813219)*, r | 450.90 (312.93) | 1.5x10^-01^ |  |  |  |
| *PAK3* *(rs10521535)*, d | 752.30 (229.63) | 1.2x10^-03^ |  |  |  |
| *PAK7* *(rs7272361)*, d | -290.02 (122.85) | 1.9x10^-02^ |  |  |  |
| *ROBO1_1_* *(rs12486635)*, r | -430.73 (135.39) | 1.7x10^-03^ |  |  |  |
| *ROBO1_2_ (rs331146)*, d | -331.37 ( 78.12) | 3.1x10^-05^ |  |  |  |
| *ROBO2_1_* *(rs1031377)*, r | 361.07 (104.80) | 6.6x10^-04^ |  |  |  |
| *ROBO2_2_* *(rs6779560)*, r | 986.29 (353.97) | 5.8x10^-03^ |  |  |  |
| *ROBO2_3_* *(rs7640201)*, d | -664.93 (188.16) | 4.8x10^-04^ |  |  |  |
| *SEMA3E_1_* *(rs1639304)*, r | 281.84 (188.58) | 1.4x10^-01^ |  |  |  |
| *SEMA3E_2_* *(rs42002)*, r | -424.86 (124.28) | 7.3x10^-04^ |  |  |  |
| *SEMA4D (rs9969727)*, r | 179.12 ( 87.16) | 4.1x10^-02^ |  |  |  |
| *SEMA5A (rs6873909)*, r | 16.38 (306.90) | 9.6x10^-01^ |  |  |  |
| *SLIT2_1_* *(rs10516357)*, d | -104.14 ( 91.55) | 2.6x10^-01^ |  |  |  |
| *SLIT2_2_* *(rs16869739)*, r | -1052.96 (377.31) | 5.7x10^-03^ |  |  |  |
| *SLIT2_3_* *(rs644607)*, r | 1368.48 (279.36) | 1.7x10^-06^ |  |  |  |
| *SLIT2_4_ (rs9992591)*, r | 429.13 (139.80) | 2.4x10^-03^ |  |  |  |
| *SLIT3_1_ (rs1432906)*, d | 335.31 ( 88.26) | 1.8x10^-04^ |  |  |  |
| *SLIT3_2_* *(rs2879135)*, r | -30.39 (241.58) | 9.0x10^-01^ |  |  |  |
| *SLIT3_3_* *(rs297838)*, r | 1030.35 (317.25) | 1.3x10^-03^ |  |  |  |
| *SRGAP3_1_* *(rs3732977)*, r | 1122.79 (316.61) | 4.6x10^-04^ |  |  |  |
| *SRGAP3_2_* *(rs557377)*, r | 833.81 (152.83) | 1.1x10^-07^ |  |  |  |
|  |  |  |  |  |  |

* Subscript is used to identify individual SNPs in interactions.

^†^ a = log-additive, d = Mendelian dominant, r = Mendelian recessive.
